# Nano-Zirconium Dioxide Catalyzed Multicomponent Synthesis of Bioactive Pyranopyrazoles That Target Cyclin Dependent Kinase 1 in Human Breast Cancer Cells

**DOI:** 10.3390/biomedicines11010172

**Published:** 2023-01-10

**Authors:** Basappa Basappa, Lisha K. Poonacha, Zhang Xi, Divakar Vishwanath, Ji-Rui Yang, Omantheswara Nagaraja, Ananda Swamynayaka, Mahendra Madegowda, Arunachalam Chinnathambi, Sulaiman Ali Alharbi, Doddahosuru Mahadevappa Gurudatt, Vijay Pandey, Nanjundaswamy Shivananju, Kwang Seok Ahn, Gautam Sethi, Peter E. Lobie, Priya Babu Shubha

**Affiliations:** 1 Laboratory of Chemical Biology, Department of Studies in Organic Chemistry, University of Mysore, Manasagangotri, Mysore 570006, India; 2 Department of Studies in Chemistry, University of Mysore, Manasagangotri, Mysore 570006, India; 3 Shenzhen Bay Laboratory, Shenzhen 518107, China; 4 Tsinghua Berkeley Shenzhen Institute, Tsinghua Shenzhen International Graduate School, Tsinghua University, Beijing 100084, China; 5 Department of Studies in Physics, University of Mysore, Manasagangotri, Mysore 570006, India; 6 Department of Botany and Microbiology, College of Science, King Saud University, P.O. Box 2455, Riyadh 11451, Saudi Arabia; 7 Department of Biotechnology, Sri Jayachamarajendra College of Engineering, JSS, Technical Institutions Campus, Mysore 570006, India; 8 KHU-KIST Department of Converging Science and Technology, Kyung Hee University, 24 Kyungheedaero, Dongdaemun-gu, Seoul 02447, Republic of Korea; 9 Department of Pharmacology, Yong Loo Lin School of Medicine, National University of Singapore, Singapore 119077, Singapore; 10 Institute of Biopharmaceutical and Health Engineering, Tsinghua Shenzhen International Graduate School, Tsinghua University, Beijing 100084, China

**Keywords:** pyranopyrazoles, CDK1, Gibbs free energy, CHEMBL, nano-zirconium dioxide

## Abstract

Small molecules are being used to inhibit cyclin dependent kinase (CDK) enzymes in cancer treatment. There is evidence that CDK is a drug-target for cancer therapy across many tumor types because it catalyzes the transfer of the terminal phosphate of ATP to a protein that acts as a substrate. Herein, the identification of pyranopyrazoles that were CDK inhibitors was attempted, whose synthesis was catalyzed by nano-zirconium dioxide via multicomponent reaction. Additionally, we performed an in-situ analysis of the intermediates of multicomponent reactions, for the first-time, which revealed that nano-zirconium dioxide stimulated the reaction, as estimated by Gibbs free energy calculations of spontaneity. Functionally, the novel pyranopyrazoles were tested for a loss of cell viability using human breast cancer cells (MCF-7). It was observed that compounds **5b** and **5f** effectively produced loss of viability of MCF-7 cells with IC_50_ values of 17.83 and 23.79 µM, respectively. In vitro and in silico mode-of-action studies showed that pyranopyrazoles target CDK1 in human breast cancer cells, with lead compounds **5b** and **5f** having potent IC_50_ values of 960 nM and 7.16 μM, respectively. Hence, the newly synthesized bioactive pyranopyrazoles could serve as better structures to develop CDK1 inhibitors against human breast cancer cells.

## 1. Introduction

**Figure 1:**
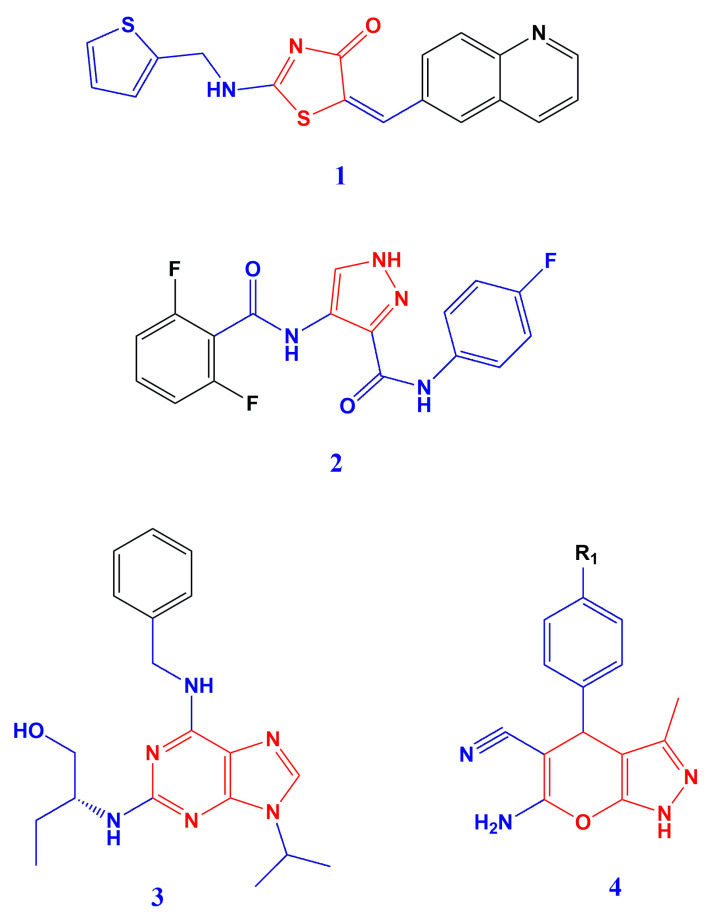
Structural evolution of newer CDK1 inhibitors. thiazolone (**1**), pyrazole (**2**), purine (**3**) and pyranopyrazole (**4**) based heterocycles were used to anchor at the CDK enzyme catalytic pocket.

The cyclin-dependent kinases (CDKs) are proteins that are involved in the control of cell cycle progression [1,2,3]. The loss of cell cycle control that results in aberrant cellular proliferation is considered a fundamental characteristic of cancer, and inhibitors of CDKs provide a method of controlling cancer progression and hence have therapeutic implications [4,5,6,7,8]. There are currently around six types of CDK inhibitors based on the enzyme binding pockets that have been designed so far and CDK1 is one of the most appealing drug targets [9]. On the basis of the co-crystal structure of 3-phosphoinositide-dependent protein kinase 1 with ATP, the inhibitors were classified as type-I inhibitors, which bind to the active conformation of the enzyme that occurs when the aspartate amino acid of the DFG motif is pointing into the ATP binding region [9]. The aspartate amino acid switches from its active site position to the inactive conformation in type-II inhibitors. An inhibitor that targets the allosteric region near the ATP-binding pocket is referred to as a type-III inhibitor. The type-IV inhibitors interact with the allosteric site, which is located away from the region where the ATP is bound. Allosteric amino acids and ATP-binding regions are known to be occupied by type-V inhibitors [10]. Type-VI inhibitors irreversibly bind to either the ATP-binding, or allosteric region of the enzyme [10]. According to published reports, an aminothiazole compound, namely RO-3306 (**1**) was found to be a type-I inhibitor that inhibits CDK1 with high potency in cancer cells compared to normal cells [11,12]. Specifically, co-crystal structural analysis of CDK1 bound to a potent ATP-competitive inhibitor compound 23 [4-(2,6-difluoro-benzoylamino)-1*H*-pyrazole-3-carboxylic acid (4-Fluoro-phenyl)-amide; **2**] infers plasticity in the CDK1 substrate binding region, when compared to CDK2 [13]. These studies were a breakthrough in developing CDK1-selective inhibitors, as the main amino acid residues that interact with compound 23 are identical in CDK1 and CDK2; resulting in a very similar activity towards these two CDKs [13,14]. There is a deeper understanding of the structural binding of purine analogues—namely roscovitine (**3**) which was designed, synthesized, and analyzed as a potent CDK inhibitor that interacts with the ATP-binding site with respect to C-8 substitution [15]. Since biogenic purine heterocycles were studied as structural analogues with pyrazolopyrimidines, it was herein attempted to synthesize newer pyranopyrazoles (**4**) with a substitution at pyrano-carbon that could be bio-isosteric with C-8 substituted purines (Figure 1) [16].

Furthermore, we report the synthesis of pyranopyrazoles via multi-component reactions (MCRs) catalyzed by recyclable nano-zirconium dioxide, which allows us to produce a high yield of products, fewer by-products as compared with conventional synthesis, and consequently reduced cost, time, and energy to synthesize, thereby allowing us to create new catalytic systems. In addition, the MCR mechanism was studied by in silico Gibbs free energy calculations, which correlates with the reaction spontaneity in the presence of nano-zirconium dioxide, and our in vitro studies revealed that the newly synthesized pyranopyrazoles could be used as a template to probe CDK1 in human breast cancer cells.

## 2. Materials and Methods

### 2.1. Synthesis of Nano-Zirconium Dioxide

Hydrated zirconyl nitrate (ZrO(NO_3_)_2_·xH_2_O (ZN), Aldrich, INDIA, purity almost 100%) and glycine (NH_2_CH_2_COOH, Mallinckrodt, St. Louis, MO, USA, purity 99.5%, Gly) were utilized as the precursors. The various results of the redox combinations (Gly:ZN) for burning were determined by utilizing the complete reducing (+9) and oxidizing (−10) valences of the precursor: Gly and ZN, respectively. As indicated by the rules of fuel science [17], for a stoichiometric redox response, the proportion of the net reducing valency of the fuel relative to the oxidizing valency of the metal nitrate ought to be solid (most extreme amount of energy delivered in the ignition cycle). Accordingly, the Gly:ZN molar proportion for the stoichiometric ignition ought to be 1:11. Also, fuel-lean (0.5 and 0.75) and fuel-rich (2) Gly:ZN precursors were applied for test planning. The determined Gly:ZN molar proportion was decided tentatively to ensure the auto-start of the ignition process, taking into account that it happens in a restricted range of fuel-to-oxidant molar proportions (above and below the stoichiometric one). The necessary amounts of starting materials were crushed in a base measure of deionized water and blended to acquire a straightforward fluid arrangement of oxidant–fuel precursor. After a lack of hydration at ca. 80 °C, when a gooey fluid was produced, the temperature was increased to ca. 250 °C. This prompted a quick, self-supporting, flameless, non-dangerous auto-ignition of the fluid, with a rapid development of a large amount of gases and the development of an undefined powder (as affirmed by XRD, not displayed here), which suggested inadequate burning. The initial idea at the start and the amounts of the resultant powders, relied upon the fuel-to-oxidant molar proportions. Consequently, these examples were used as crude powders. Therefore, they were calcined in air, at 55 °C for 4 h at barometric pressure, to eliminate residual unreacted starting materials (if any) and additionally, results of their disintegration gave unadulterated, and very much solidified oxides which were examined by XRD.

### 2.2. Chemistry

The progress response was determined utilizing thin layer chromatography (TLC). Analytical TLC was performed on precoated Merck silica gel 60 F254 plates (INDIA) using ethyl acetate and hexane as eluent, and spots were detected under UV light. ^1^H NMR and ^13^C NMR spectra were recorded on an Agilent NMR instrument in DMSO as the solvent (Santa Clara, CA, USA). Chemical shifts were expressed in ppm comparative to TMS. Mass spectra were recorded on an Agilent LC-MS (Santa Clara, CA, USA). All solvents and reagents were reagent grade.

#### 2.2.1. General Procedure for Preparation of Newer Pyranopyrazole Derivatives **5**(**a**–**o**)

A mixture of aryl aldehyde (**1**) (1 mmol), substituted hydrazine hydrate (**2**) (1 mmol), ethyl acetoacetate (**3**) (1 mmol), malononitrile (**4**) (1 mmol), and nano-zirconium dioxide were stirred in water/ethanol (1:1) at room temperature for about 30–60 min (Table 1 and Table 2). After completion of the reaction, catalyst was regained by filtration. Further, the filtrate was worked up using ethyl acetate (25 mL × 3), and the solvent evaporated under high pressure vacuum. The crude products were purified through a column chromatography technique to obtain the desired compounds **5**(**a**–**o**). Novel compounds were confirmed by ^1^H, ^13^C NMR, mass spectroscopy and reported molecules were confirmed by comparing their melting points with literature data.

#### 2.2.2. Characterization of 6-Amino-3-methyl-4-(4-(pyrimidin-5-yl)phenyl)-1,4-dihydropy-rano [2,3-c] Pyrazole-5-carbonitrile (**5a**)

Brown solid; MP: 196–198 °C; 70% yield; ^1^H NMR (500 MHz, DMSO): *δ* 10.01 (s, 1H), 8.96 (s, 1H), 8.77 (s, 2H), 7.70 (d, *J* = 7.5 Hz, 2H), 7.65 (s, 2H), 7.14 (d, *J* = 7.0 Hz, 2H), 5.51 (s, 1H), 2.30 (s, 3H); MS: 330.34, m/z = 331.12 [M+H]^+^.

#### 2.2.3. Characterization of 6-Amino-4-(3-(6-fluoro-5-methylpyridin-3-yl)phenyl)-3-methyl-1,4-dihydropyrano [2,3-c] Pyrazole-5-carbonitrile (**5b**)

Yellow solid; MP: 214–215 °C; 63% yield; ^1^H NMR (500 MHz, DMSO): *δ* 10.02 (s, 1H), 8.34 (d, *J* = 2.5 Hz, 1H), 7.87–7.85 (m, 1H), 7.65 (s, 2H), 7.60 (dt, *J* = 7.4, 2.4 Hz, 1H), 7.42 (t, *J* = 7.4 Hz, 1H), 7.23 (d, *J* = 7.3 Hz, 1H), 7.11 (t, *J* = 2.1 Hz, 1H), 5.50 (s, 1H), 2.56 (s, 3H), 2.30 (s, 3H); ^13^C NMR (100 MHz, DMSO): *δ* 163.24, 161.85, 160.88, 155.57, 146.15, 143.13, 142.98, 141.19, 136.65, 134.80, 130.96, 127.90, 126.5, 125.99, 120.47, 120.12, 119.7, 97.80, 57.13, 36.41, 14.06, 9.91; MS: 361.38, m/z = 362.14 [M+H]^+^.

#### 2.2.4. Characterization of 6-Amino-3-methyl-4-(3-(pyridin-4-yl)phenyl)-1,4-dihydropyrano [2,3-c] Pyrazole-5-carbonitrile (**5c**)

Dark yellow solid; MP: 245–247 °C; 78% yield; ^1^H NMR (600 MHz, DMSO): *δ* 9.93 (s, 1H), 8.84 (d, *J* = 2.4 Hz, 1H), 8.56 (dd, *J* = 7.5, 2.6 Hz, 1H), 7.82 (dt, *J* = 7.5, 2.5 Hz, 1H), 7.70 (d, *J* = 7.5 Hz, 2H), 7.65 (s, 2H), 7.31 (t, *J* = 7.4 Hz, 1H), 7.14 (d, *J* = 7.2 Hz, 2H), 5.47 (s, 1H), 2.30 (s, 2H); ^13^C NMR (100 MHz, DMSO): *δ* 161.82, 155.59, 149.16, 148.35, 145.31, 140.85, 136.37, 136.17, 134.68, 129.82, 128.91, 127.69 126.42, 124.52, 121.49, 97.99, 57.20, 36.04, 9.89; MS: 329.36, m/z = 330.17 [M+H]^+^.

#### 2.2.5. Characterization of 6-Amino-4-(3-(6-chloro-5-methylpyridin-3-yl)phenyl)-3-methyl-1,4-dihydropyrano [2,3-c] Pyrazole-5-carbonitrile (**5d**)

Brown solid; MP: 212–214 °C; 82% yield; ^1^H NMR (600 MHz, DMSO): *δ* 10.02 (s, 1H), 8.60 (d, *J* = 2.4 Hz, 1H), 7.89–7.87 (m, 1H), 7.60 (dt, *J* = 7.5, 2.5 Hz, 1H), 7.42 (t, *J* = 7.5 Hz, 1H), 7.23 (dtd, *J* = 7.5, 2.5, 1.0 Hz, 1H), 7.11 (td, *J* = 2.5, 1.0 Hz, 1H), 5.51 (s, 1H), 2.53 (s, 3H), 2.30 (s, 3H); ^13^C NMR (100 MHz, DMSO): *δ* 161.83, 155.54, 150.32, 146.18, 145.61, 138.68, 136.37, 135.70, 132.87 130.18, 128.21, 126.56, 126.06, 121.41, 97.74, 57.13, 36.40, 19.09, 9.87; MS: 377.83, m/z = 378.14 [M+H]^+^.

#### 2.2.6. Characterization of 6-Amino-3-methyl-4-(4-(pyridin-4-yl)phenyl)-1,4-dihydropyra-no [2,3-c] Pyrazole-5-carbonitrile (**5e**)

Brown solid; MP: 232–234 °C; 80% yield; ^1^H NMR (600 MHz, DMSO): *δ* 9.93 (s, 1H), 8.79 (d, *J* = 7.4 Hz, 2H), 7.77 (d, *J* = 7.4 Hz, 2H), 7.70 (d, *J* = 7.5 Hz, 2H), 7.65 (s, 2H), 7.14 (d, *J* = 7.2 Hz, 2H), 5.47 (s, 1H), 2.30 (s, 3H); ^13^C NMR (100 MHz, DMSO): *δ* 161.76, 155.56, 150.96, 147.36, 146.46, 136.33, 136.205, 128.94, 127.63, 121.67, 121.36, 97.80, 57.09, 36.04, 9.82; MS: 329.36, m/z = 330.16 [M+H]^+^.

#### 2.2.7. Characterization of 6-Amino-4-(4-(6-fluoro-5-methylpyridin-3-yl)phenyl)-3-methyl-1,4-dihydropyrano [2,3-c] Pyrazole-5-carbonitrile (**5f**)

Yellow solid; MP: 268–269 °C; 79% yield; ^1^H NMR (600 MHz, DMSO): *δ* 10.02 (s, 1H), 8.26 (d, *J* = 2.5 Hz, 1H), 7.70 (d, *J* = 7.4 Hz, 2H), 7.65 (s, 2H), 7.14 (d, *J* = 7.2 Hz, 2H), 6.98–6.96 (m, 1H), 5.50 (s, 1H), 2.56 (s, 3H), 2.30 (s, 3H); ^13^CNMR (100 MHz, DMSO): *δ* 169.58, 162.28, 160.93, 154.76, 144.42, 142.30, 142.15, 140.41, 135.63, 134.43, 128.17, 126.93, 120.77, 119.33, 119.00, 97.40, 56.94, 35.85, 13.99, 9.80; MS: 361.38, m/z = 362.18 [M+H]^+^.

#### 2.2.8. Characterization of 6-Amino-1-(4-chlorophenyl)-3-methyl-4-(3-(pyrimidin-5-yl)phenyl)-1,4-dihydropyrano [2,3-c] Pyrazole-5-carbonitrile (**5g**)

MP: 242–243 °C; ^1^H NMR (400 MHz, DMSO): *δ* 9.15 (tdd, *J* = 16.3, 10.4, 6.1 Hz, 4H), 7.95–7.63 (m, 4H), 7.29 (dd, *J* = 14.7, 8.2 Hz, 3H), 6.98 (s, 2H), 4.37 (s, 1H), 2.33 (s, 3H);^13^C (100 MHz, DMSO): *δ* 166.23, 161.44, 159.41, 157.98, 157.90, 155.45, 147.54, 146.49, 143.14, 140.19, 136.55, 134.91, 134.54, 133.62, 133.03, 132.21, 131.93, 130.57, 128.77, 127.81, 120.29, 114.77, 113.32, 107.47, 106.57, 57.21, 18.32, 13.83; MS: 440.17, m/z = 441.11 [M+H]^+^.

### 2.3. Gibbs Free Energy Calculation

The geometry of all the plausible intermediates and products were constructed using GaussView software [18] and fully optimized with the aid of the density function theory method (DFT) by employing the B3LYP functional [19] and the LanL2DZ basis set without any symmetry constraints. The optimization calculations were performed in the gaseous phase using the Gaussian 09W software package [20].

### 2.4. Cell Viability Assay

We obtained MCF-7 cells from Procell Life Science and Technology. A humidified atmosphere of 5% CO_2_ was maintained at 37 °C for the culture of MCF-7 cells (2000) in MEM or Leibovitz’s L-15 medium enriched with 2% FBS [21,22,23,24]. DMSO was used to prepare a stock solution of pyranopyrazoles, and the stock solution was then diluted with culture medium to achieve the desired concentration. A series of compounds were applied to MCF-7 cells in 96-well plates for 12 h followed by 72 h of treatment with or without pyranopyrazoles at concentrations of 0, 0.01, 0.1, 10, 100, and 1000 µM. Incubation with AlamarBlue assay reagent was performed for a further 4 h. According to the established protocol, the IC_50_ values of compounds were determined in the absence and presence of pyranopyrazoles.

### 2.5. In Silico Mode-of-Action Analysis

The mode-of-action of pyranopyrazoles that inhibit the proliferation of human breast cancer cells was investigated using the CHEMBL database [25,26,27,28,29,30,31,32,33,34,35]. The pyranopyrazole core-structure was used to retrieve CHEMBL database bioactivity profiles via a ligand similarity search which comprises the organism, genes, microbes, viruses, and other bioassays as classifications and rankings.

### 2.6. Kinase Assay

Promega’s Kinase-Glo luminescence assay was used for detection of CDK1 activity. IgG was used as a negative control, and 1 mg of CDK1 antibodies were used to immunoprecipitate 1 mg of protein from the total cell lysate. At 41 °C, the reaction mixture was incubated for three hours and subsequently, beads were washed three times with lysis buffer, following overnight incubation at 41 °C. The reactant beads were added to 10 mL kinase reaction buffer containing 0.2 mM ATP, 2 mM DTT, 0.1 mg/mL BSA, 20 mM MgCl_2_, and 40 mM Tris-HCl, and beads were resuspended for 30 min at room temperature with a CDK1/2 specific substrate (p53). A total of 10 µl of ADP-GLO reagent and 10 mL of kinase detection reagent were added to the reaction for 40 min at room temperature and 5 min at room temperature, respectively. Each experiment consisted of loading 1 mL of the mixture and analyzing it with CDK-1 antibodies.

### 2.7. In Silico Bioinformatic Analysis

A molecular docking study was performed with the synthesized compound and CDK1. Docking the lead compound **5b** and co-crystallized ligand was carried out using the Scripps Research Institute’s AutoDockTools (ADT) (v1.5.7) [13]. The X-ray crystallographic structure of CDK1 in complex with the ligand (PDB code: 4Y72 [29]) was downloaded from the Protein Data Bank (www.rcsb.org-accessed on 6 June 2020) and prepared for docking calculations. The AutoDock protocol was followed to prepare the pdbqt file for the receptor by deleting the heteroatoms and adding polar hydrogen atoms. The receptor was fixed, and docking of compound **5b** was performed in the catalytic site of the CDK1 enzyme. With an initial population of 150 randomly placed individuals, a maximum number of 2,500,000 energy evaluations, a mutation rate of 0.02, a crossover rate of 0.80, and 10 docking runs, the empirical-free energy function and the Lamarckian Genetic Algorithm were used to perform molecular docking with the macromolecule. The grid with a size of 60 × 60 × 60 was placed in the center of the active site. The built-in clustering analysis was used to process the predicted binding poses for each compound, with the confirmation of the lowest energy with respect to the largest cluster chosen as the representative. BIOVIA Discovery Studio Visualizer (v21.10.20298) [36] and PyMOL (v2.5.2) [37] were used to examine the modeled structure.

### 2.8. Statistical Analysis

The data were analyzed by Student’s *t*-test and *p* < 0.05 was considered statistically significant (GraphPad Prism 5.0; GraphPad Software, La Jolla, CA, USA).

## 3. Results

### 3.1. Synthesis and Characterization of Nano-Zirconium Dioxide

**Figure 2:**
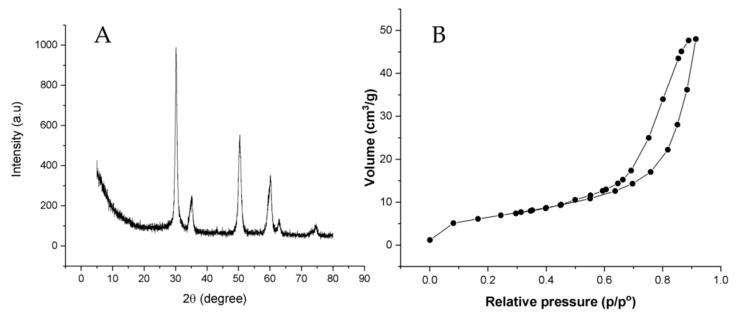
PXRD pattern (**A**) and N_2_ adsorption–desorption isotherms (**B**) of nano-ZrO_2_.

X-ray diffraction patterns of the synthesized nano-ZrO_2_ showed sharp peaks at 2 theta values of 30°, 35°, 51°, 60°, 63° corresponding to (101), (002), (200), (211), (202). This comprises of a Cu target that emits Cu Kα radiation at 40 mA and 40 kV, respectively, with a current and voltage of 40 mA and 40 kV. The XRD patterns were created with a scanning speed of 2°/min and 2° of rotation ranging from 5 to 80 degrees. All the reflections of the XRD patterns were indexed to the standard pattern of the pure cubic phase of zirconia. This reveals that the zirconia sample synthesized by the combustion method produced a cubic structure. Diffraction peaks in Figure 2A with 2θ value 2.96, 2.56, 1.81, 1.54, 1.48, 1.28, 1.17, 1.14, 1.04, 0.98, 0.90, 0.86, 0.85, 0.85 originate from the crystal planes (111), (200), (220), (311), (222), (400), (331), (420), (422), (511), (440), (531), (600), (620) of cubic zirconia, respectively. Further, the surface area of ZrO_2_ was obtained by the N_2_ adsorption technique. The isotherm of ZrO_2_ was found to be type IV and H3 hysteresis loop, which is characteristic of a mesoporous structure with a surface area of 22.825 m^2^/g. The total pore volume and mean pore diameter was about 0.0742 cm^3^/g, and 13.011 nm, respectively (Figure 2B).

### 3.2. Synthesis of Pyranopyrazoles

**Scheme 1:**
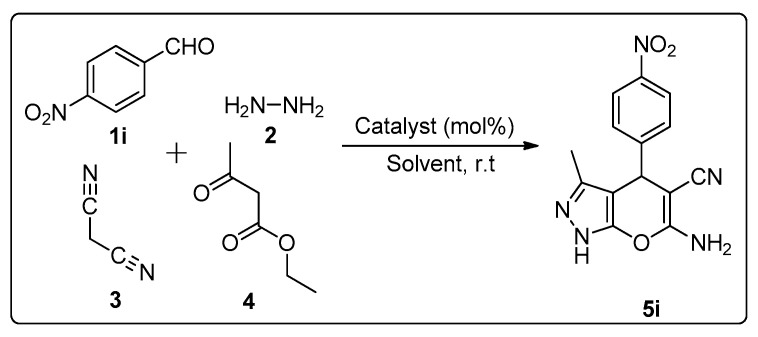
Optimization of reaction conditions.

**Scheme 2:**
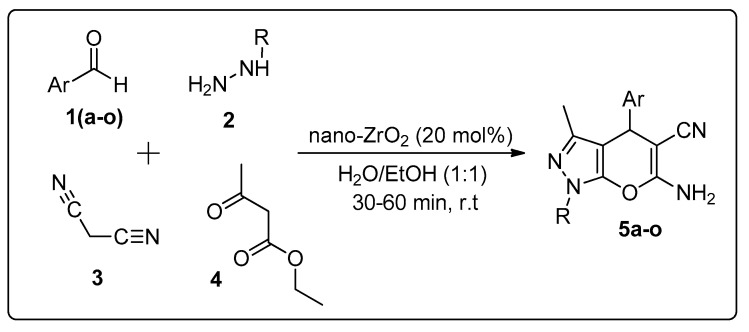
General schematic representation for the synthesis of pyranopyrazoles.

Using MCR, the synthesis of pyranopyrazoles were carried out in the presence of a nano-ZrO_2_ catalyst. The five-component reaction was primarily performed between 4-nitro benzaldehyde (**1i**), hydrazine hydrate (**2**), malononitrile (**3**), ethyl acetoacetate (**4**), and nano-ZrO_2_ (10 mol%) in water at room-temperature for 30 min and afforded **5i** with 38% yield (Scheme 1 and Scheme 2, Table 1 and Table 2, entry-1). Further, the reaction was carried out using different solvents like ethanol and a mixture of water–ethanol (1:1) for about 30 min, it was found that the percentage yield (53%) was slightly increased in a water–ethanol mixture (Table 1, entry-3). The same reaction was carried out by altering the amount of catalyst to 20 mol% in a water–ethanol mixture for about 50 min, and a significant yield (75%) was observed (Scheme 1, Table 1, entry-6). Moreover, increasing the amount of catalyst has no effect on the percentage yield. The optimization reaction conditions, and the outcomes are summarized in Table 1 and Table 2. The new compounds were characterized using NMR and Mass sepctral analysis (supplementary data).

**Scheme 3:**
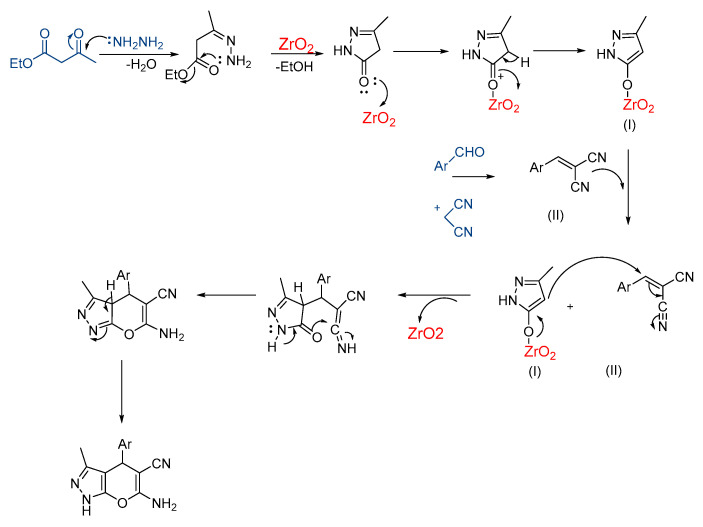
Proposed mechanism for the multi-component synthesis of pyranopyrazoles.

A plausible reaction mechanism for this condensation is shown in Scheme 3. In the synthesis of pyranopyrazoles nano-ZrO_2_ acts as both Lewis acid and base. Initially, a pyrazolone derivative was formed by the condensation reaction of ethyl acetoacetate and substituted hydrazine hydrate. ZrO_2_ accepts an electron pair from the oxygen of the carbonyl group and acts as a Lewis acid, this enables the reaction between ethyl acetoacetate and hydrazine hydrate. The Lewis base site of ZrO_2_ enables malononitrile to generate an active methylene group. Thus, the presence of an active methylene group initiated the Knoevenagel condensation reaction between benzaldehyde and malononitrile forming arylidene malononitrile. Further, a Michael addition reaction occurred between pyrazolone and arylidene malononitrile which was followed by cyclization and tautomerization to form pyranopyrazole.

### 3.3. In Silico Mechanistic Studies of Pyranopyrazole Products

**Figure 3:**
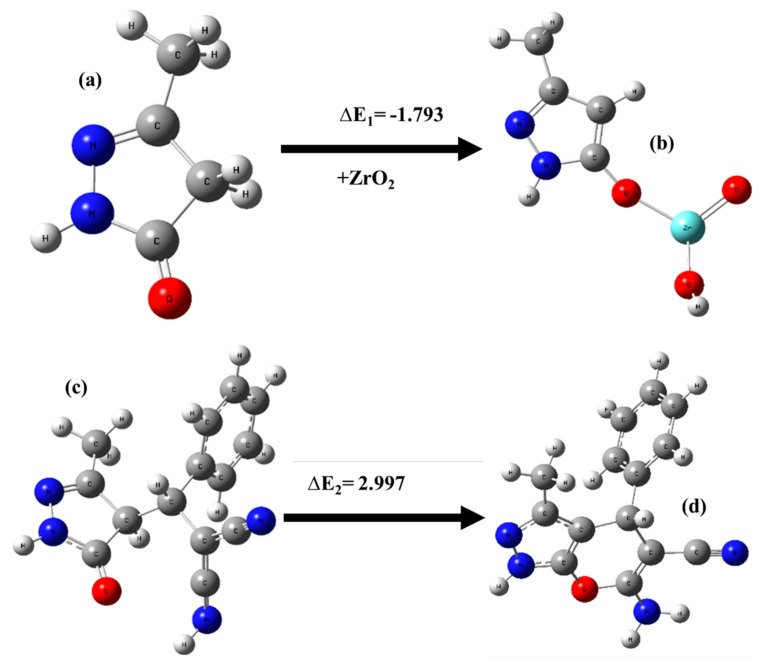
Computed intermediate reaction path and energy difference ∆E in cal/mole.

The minimized structures of the intermediates and products were validated by computing fundamental harmonic vibrational analysis at the same level of theory. For mechanistic clarification, Gibb’s free energy calculations for intermediate zirconium complexes were chosen. The ZrO_2_ makes the coordination complex with the ligand. It reacts with the pyrazolone ring, forming a Zr-O bond quickly, which is stabilized and attains a lower energy intermediate with a ∆E_1_ value of −1.793 kcal/mol. Further, the reaction between pyrazolone and arylidene malononitrile occurs by cyclization to form pyranopyrazole with an ∆E_2_ value of 2.997 kcal/mol. Figure 3 shows the optimized geometries and intermediate energy pathway. Based on intermediate reduced Gibbs free energy through (a) to (b), we can deduce that the reaction occurs spontaneously when the monodentate ligand forms nano-Zirconium dioxide coordination complexes.

### 3.4. Effect of Pyranopyrazoles on MCF-7 Cell Viability

**Figure 4:**
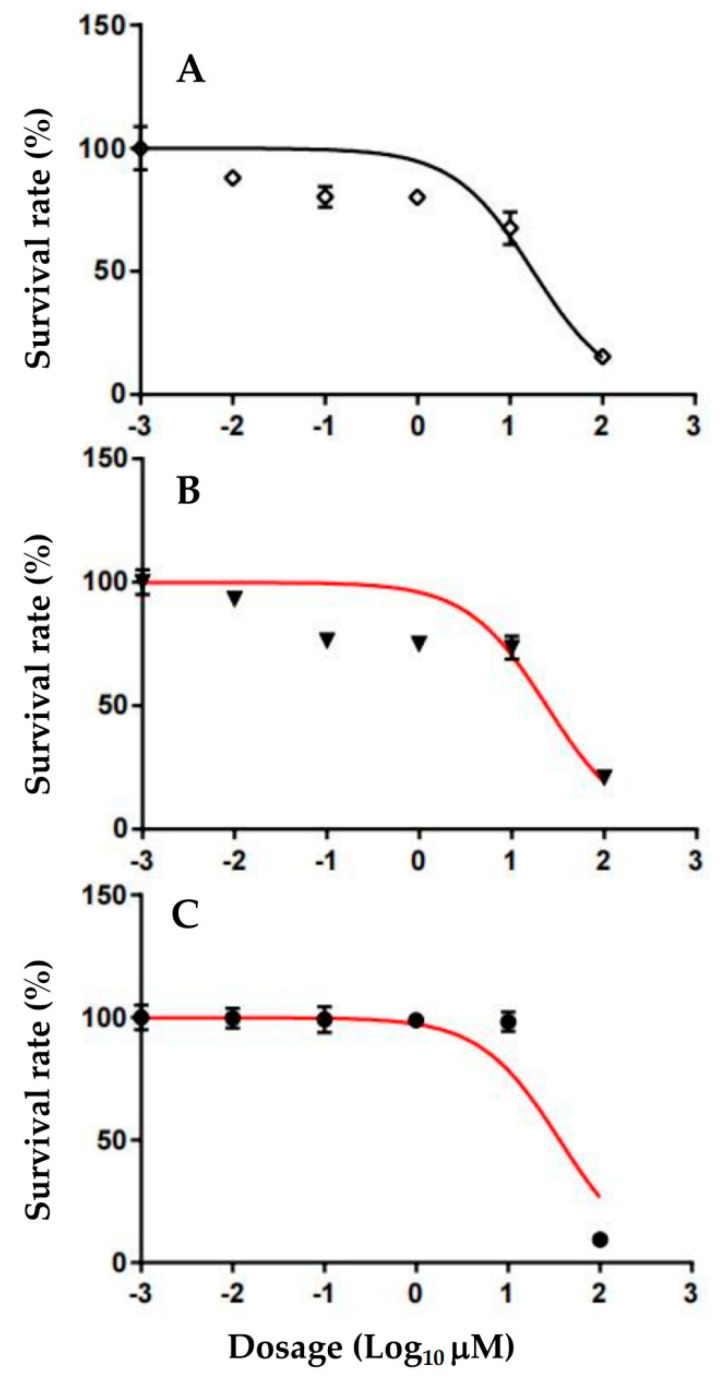
Loss of MCF-7 cell viability produced by compounds **5b 5d**, and **5f**. MCF-7 cells were exposed to **5b** (**A**), **5d** (**B**), and **5f** (**C**) for 72 h and the viability of cells was analyzed by Alamar Blue assays. The results are presented as mean ± S.E.M. of triplicate determinations.

Since 4-arylazo-3,5-diamino-1H-pyrazoles were observed to target CDKs in human breast cancer (MCF-7) cells, the effect of pyranopyrazoles on MCF-7 cell viability was determined using Alamar Blue assays [38,39,40,41,42,43,44]. Tested compounds (**5b**, **5d**, and **5f**) with different concentrations (0, 0.01, 0.1, 10, and 100 µM) showed a dose–dependent decrease in the viability of MCF-7 cells (Figure 4). Other compounds tested did not produce a significant loss of cell viability in MCF-7 cells. Compound **5b**, bearing a 3-pyridyl-4-fluoro-5-methyl ring, generated a superior IC_50_ value of 17.23 µM, when compared to other structurally related compounds.

Furthermore, we tested the most active compounds such as **5b**, **5c**, **5e**, and **5f** for the ability to inhibit human breast cancer cells’ (T47D, BT-474, SKBR3, and MDA-MB-231) proliferation (refer supplementary data). Around 2000 cells per well seeded overnight were treated with the compounds with 2% FBS conditioned medium and incubated for 3 days. The analysis of the IC_50_s of lead pyranopyrazoles revealed that the compounds could inhibit the proliferation of BT-474 cells effectively (Table 3). The tested pyranopyrazoles failed to inhibit the human breast derived normal cells.

### 3.5. In Silico Mode-of-Action Analysis of Compound ***5b***

**Figure 5:**
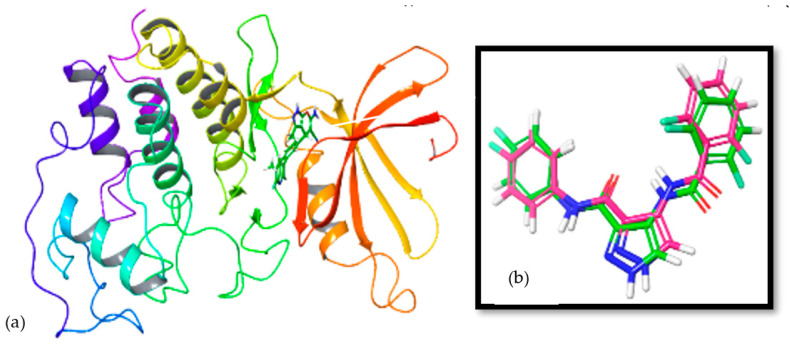
Cartoon representation of the CDK1 and compound **5b** (**a**). 3D representation of the predicted binding mode (in green) and experimental structure (in magenta) of co-crystallized ligand (**b**).

**Figure 6:**
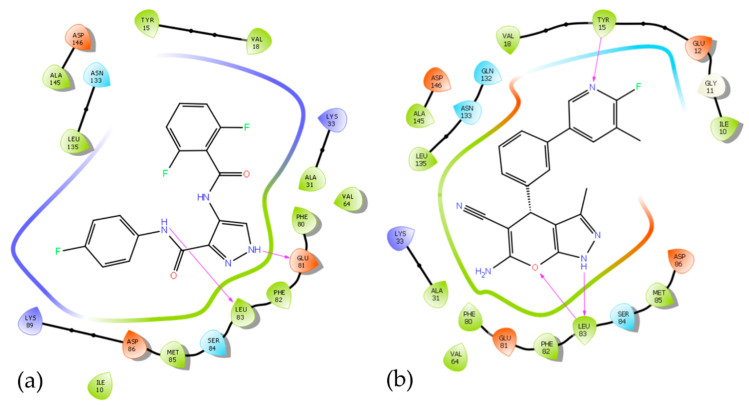
2D Interaction diagram of ligand (**a**) and **5b** (**b**)with CDK1.

**Figure 7:**
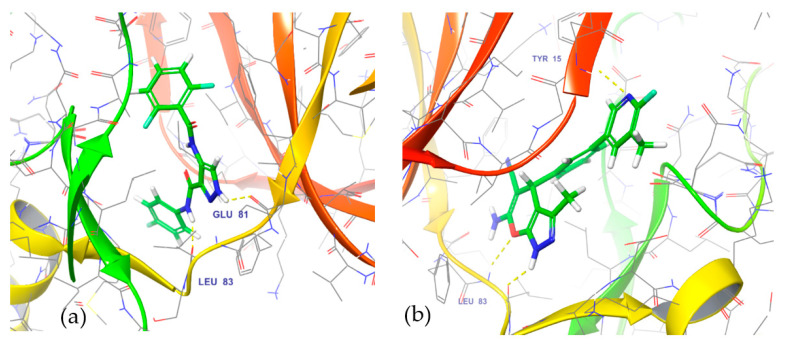
3D interaction diagram of ligand (**a**) and **5b** (**b**) with the amino acid residues of CDK1.

In silico mode-of-action analysis was performed for compound **5b** using the latest version of CHEMBL as described by Yang et al. [45]. For this purpose, the smile format of compound **5b** was added into the similarity searching engine of CHEMBL, which resulted in 2,157,379 compounds of proportionate similarity, choice of organism, cell type, and 14,855 predicted human targets [46]. The analysis of the results sheet identified CDK1 as a target for compound **5b** as the top ranking. Therefore, in silico bioinformatics was performed to determine the binding mode of compound **5b**; the employed ADT parameters and protocol were validated using the available experimental data. The binding mode of the co-crystallized ligand in the complex with CDK1 (PDB code: 4Y72) was predicted and compared to the experimental structure (Figure 5). A comparison of the predicted docked structure with the corresponding resolved crystal structure revealed that the ADT with the employed parameters correctly predicted the binding mode of the co-crystallized ligand inside the active site of CDK1, forming two essential hydrogen bonds with GLU81 and LEU83 (Figure 6). The binding mode and affinity of the synthesized compound **5b**, with the CDK1 active site was then investigated using molecular docking. The calculated docking score for the synthesized compound was −10.54 kcal/mol with three hydrogen bonds compared to the co-crystallized ligand (−9.3 kcal/mol), indicating that CDK1 inhibition is a plausible mechanism explaining the anticancer activity observed with the synthesized compound **5b**. Compound **5b** exhibited a higher binding affinity with CDK1 than the co-crystallized ligand, indicating a specific interaction of the nitrogen atom with the amino acid residue TYR15 (Figure 7).

### 3.6. In Vitro Inhibition of Lead Cyclin Dependent Kinase 1 by Pyranopyrazoles

**Figure 8:**
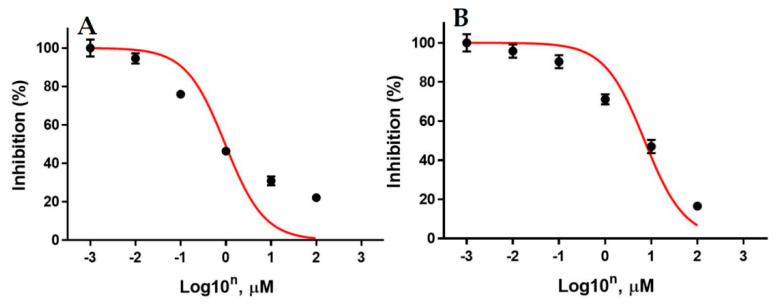
In vitro inhibition of CDK1 by compounds **5b** (**A**) and **5f** (**B**). The inhibitory effect of pyranopyrazoles on CKD1 measured using ADP−Glo™ reagent. The CDK1 reaction was performed in 1X reaction buffer and the compounds were added to the plates and incubated for 20 min, followed by the addition of ATP/substrate solution to initiate the reaction. CDK1 was added and the reaction was incubated for 40 min. We used positive controls without any compounds to calculate the activity of the kinase at 100%. To determine 0% kinase activity, negative controls were used since they did not contain any compound or enzyme. TM313 specifies the method of dispensing ADP−GloTM Kinase Assay reagents.

Since the ADP-GloTM Kinase Assay [47] uses multiple enzymes as components, it should not generate a high rate of interference or false responses when screening pyranopyrazoles anti-CDK1 activity. It is also possible to determine the kinetic parameters of CDK1 by using ADP-Glo since it can be used with a wide range of ATP and substrate concentrations. Based on the luminescence units for compounds **5b** and **5f**, Figure 8 illustrates the standard curves generated for inhibition of cdk1 activity by pyranopyrazoles at different concentrations. According to the materials and methods, equal volumes of ADP-Glo reagent were added to each well, incubated for 40 min at room temperature, and then kinase detection reagent was added. It was found that both lead molecules **5b** and **5f** had potent IC_50_ values, which were 960 nM and 7.16 μM, respectively. The results from our study are in agreement with those from PHA-793887 [48], a pyranopyrazole that mainly inhibits CDK1 with an IC_50_ value of 60 nM, by binding to the enzyme’s adenine pocket through the heterocyclic moiety (Figure 8).

## 4. Conclusions

An improved method for synthesizing pyranopyrazoles has been developed using nano-zirconium oxide. The first application of a nano-catalyst for heterocycle generation was proposed by using the in silico approach. The catalytic activity of CDK1 was significantly inhibited by two of the compounds tested. Additionally, these compounds inhibit the proliferation of human breast cancer cells in vitro. As a result of our report, we have provided new structures that can be used to develop molecules that target CDK1 in human breast cancer models.

## Figures and Tables

**Table 1:** Optimization of reaction conditions in the synthesis of pyranopyrazoles.

Entry	Solvent	Amount of Catalyst (mol%)	Time (min)	Yield (%)
1	H_2_O	10	30	38
2	EtOH	10	30	45
3	H_2_O:EtOH (1:1)	10	30	53
4	H_2_O:EtOH (1:1)	10	50	62
5	H_2_O:EtOH (1:1)	20	30	68
6	H_2_O:EtOH (1:1)	20	50	75

**Table 2:** Physical data and cytotoxicity data of 6-amino-4-aryl-3-methyl-1,4-dihhydropyrano [2,3-*c*] pyrazole-5-carbonitriles.

Entry	Aldehyde	Product	Time (min)	Melting Point (°C)	MCF-7 IC_50_ (µM)
1	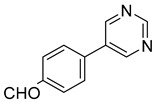 **1a**	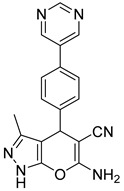 **5a**	60	196–198 ^b^	55
2	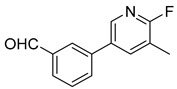 **1b**	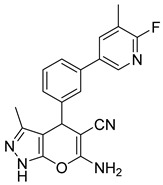 **5b**	50	214–215 ^b^	17
3	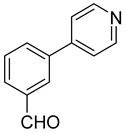 **1c**	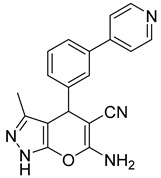 **5c**	30	245–247 ^b^	ND
4	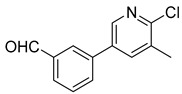 **1d**	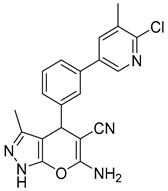 **5d**	50	212–214 ^b^	38.89
5	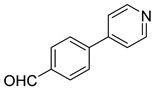 **1e**	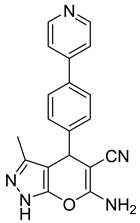 **5e**	45	232–234 ^b^	>100
6	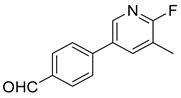 **1f**	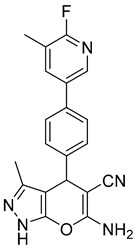 **5f**	60	268–269 ^b^	23.79
7	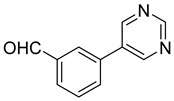 **1g**	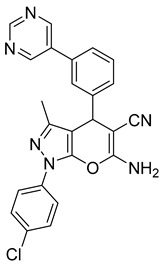 **5g**	50	242–243	ND
8	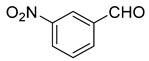 **1h**	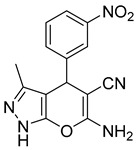 **5h**	40	232–234	ND
9	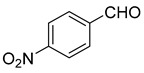 **1i**	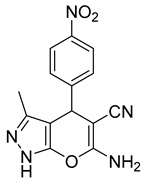 **5i**	35	249–250	ND
10	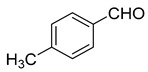 **1j**	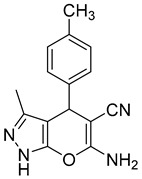 **5j**	60	196–198 ^a^	ND
11	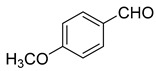 **1k**	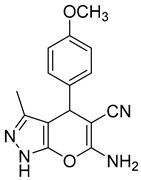 **5k**	45	211–213	ND
12	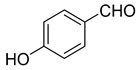 **1l**	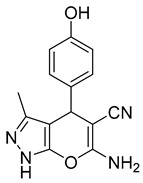 **5l**	35	224–226 ^a^	ND
13	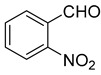 **1m**	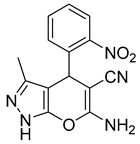 **5m**	50	241–242	ND
14	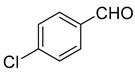 **1n**	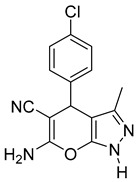 **5n**	40	233–234	ND
15	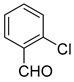 **1o**	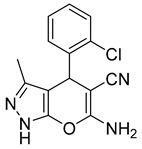 **5o**	50	246–248	ND
Olaparib					3.28

^a^ Melting point compared with literature. ^b^ Melting point observed experimentally.

**Table 3:** Cytotoxicity data of pyranopyrazoles against TNBC and MCF-10A cells.

Compound Name	IC50 (μM) (Alamar Blue)				
	T47D	BT-474	SKBR3	MDA-MB-231	MCF-10A
**5b**	27.48	23.66	>100	13.47	90.52
**5c**	33.71	21.28	69.38	14.95	88.25
**5e**	>100	50.17	>100	22.07	>100
**5f**	45.79	15.23	>100	19.1	>100

## Data Availability

The supplementary data could be considered as supportive data of the results of this article.

## Supplementary Materials

The following supporting information can be downloaded at: https://www.mdpi.com/article/10.3390/biomedicines11010172/s1.

## Sample Availability

The synthesized compounds are available from Basappa’s Laboratory.
