# Supplementary material for: Nano-Zirconium Dioxide Catalyzed Multicomponent Synthesis of Bioactive Pyranopyrazoles That Target Cyclin Dependent Kinase 1 in Human Breast Cancer Cells
Source: Biomedicines. 2023 Jan 10;11(1):172. doi: 10.3390/biomedicines11010172 (PMC9856062; doi:10.3390/biomedicines11010172)
Supplement: Supplementary file 1 [file biomedicines-11-00172-s001.zip › biomedicines-2144955-supplementary.pdf]

Nano-zirconium dioxide catalyzed multi-component synthesis of bioactive pyranopyrazoles that targets cyclin dependent kinase 1 in human breast cancer cells

Lisha K. Poonacha, Zhang Xi, Ji-Rui Yang, Omantheswara Nagaraja, Divakar V, Ananda Swamynayaka, Mahendra Madegowda, Arunachalam Chinnathambi, Sulaiman Ali Alharbi, Doddahosuru Mahadevappa Gurudatt, Vijay Pandey, NanjundaSwamy Shivananju, Kwang Seok Ahn, Gautam Sethi, Priya Babu Shubha, Peter E. Lobie, Basappa Basappa

Supplementary data

Spectral data

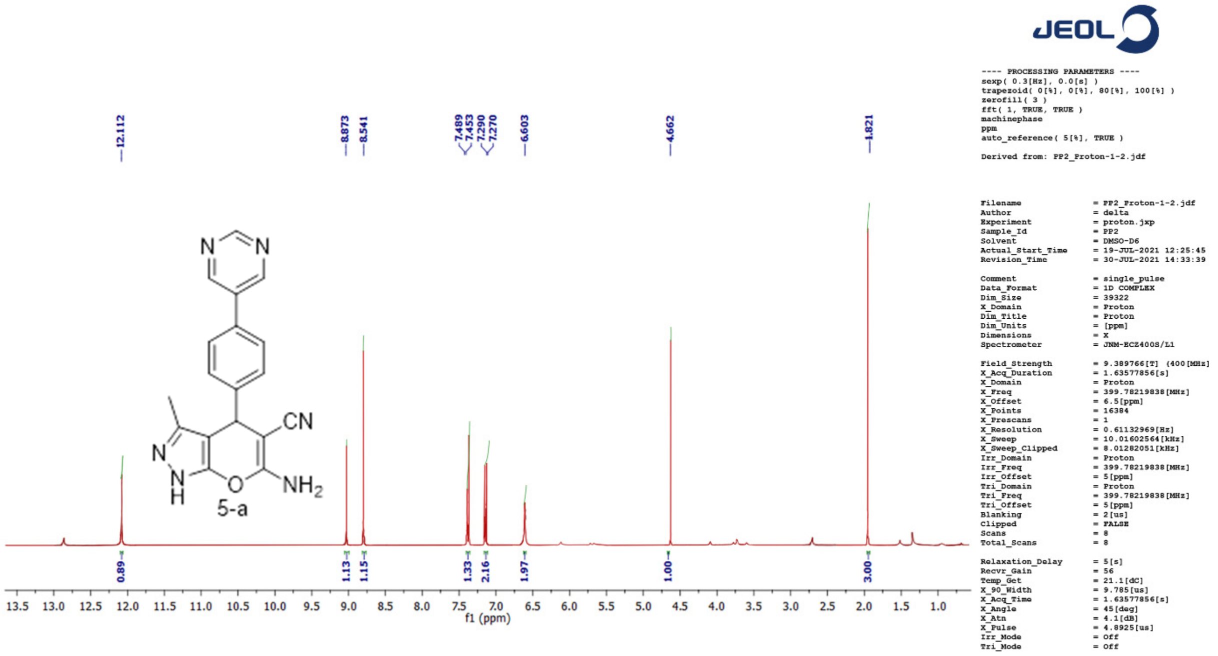

^{1}H NMR of 5-a

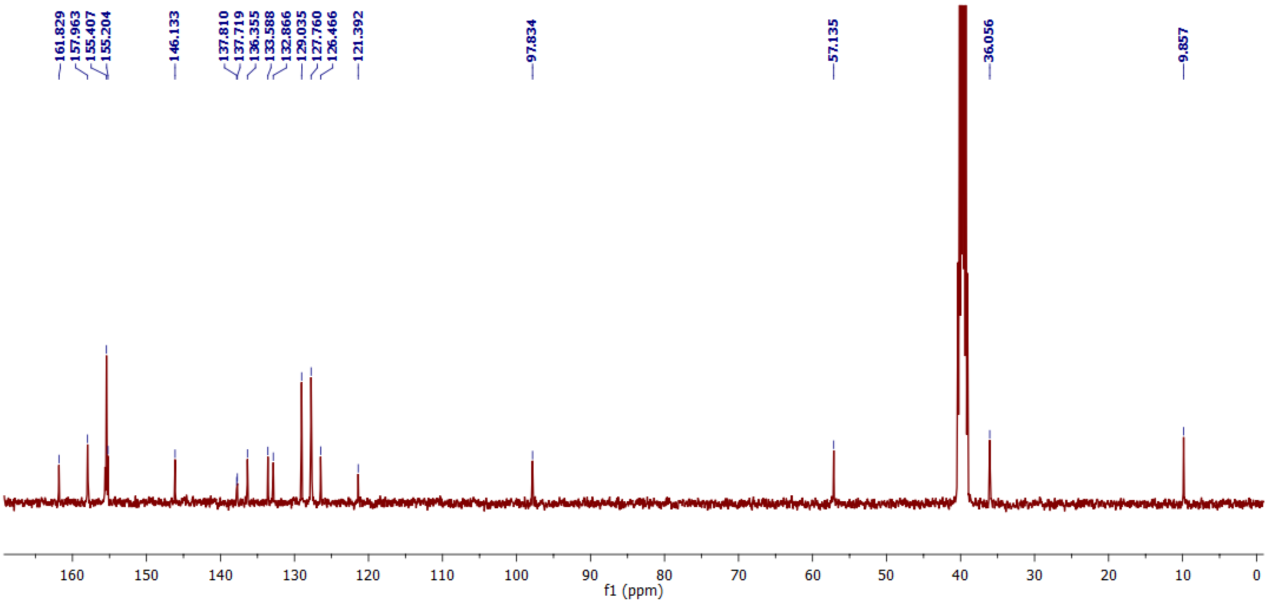

^{13}C NMR of 5-a

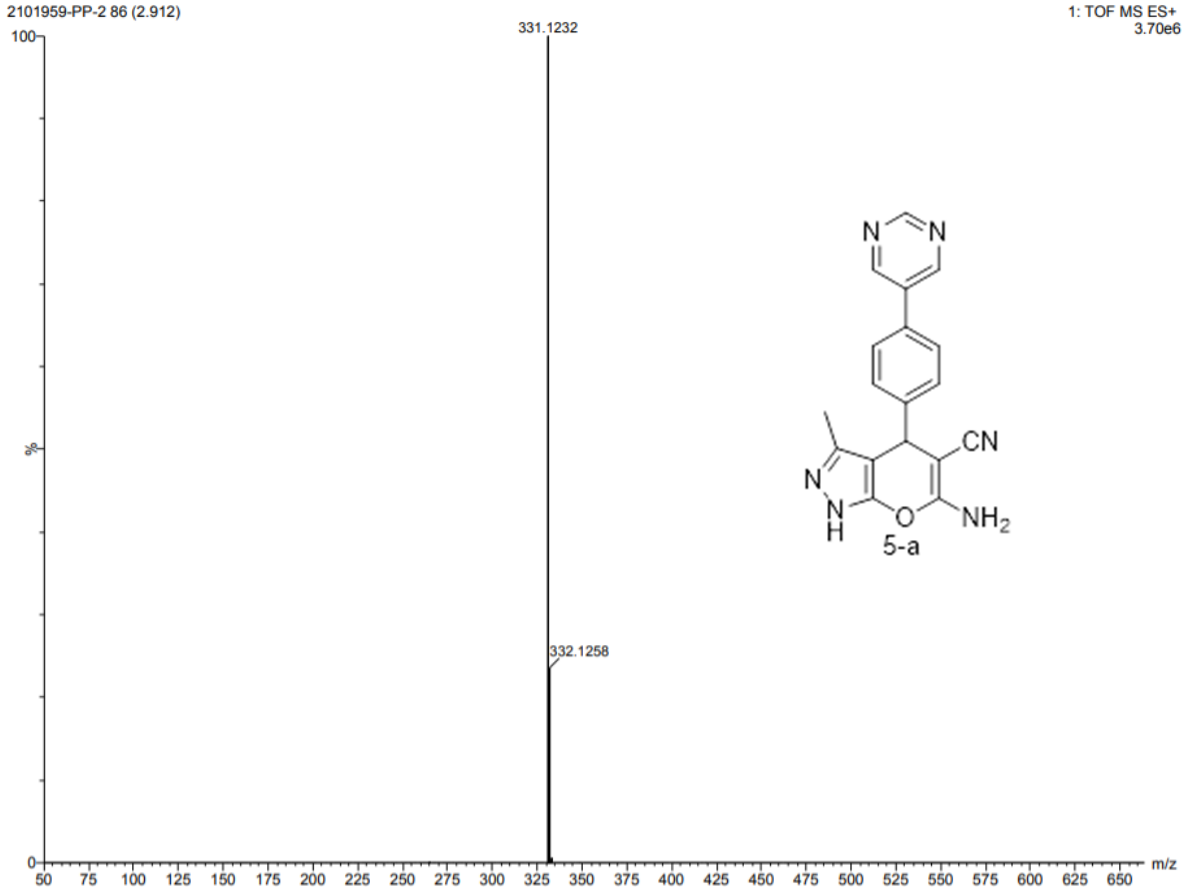

Mass spectra of 5-a

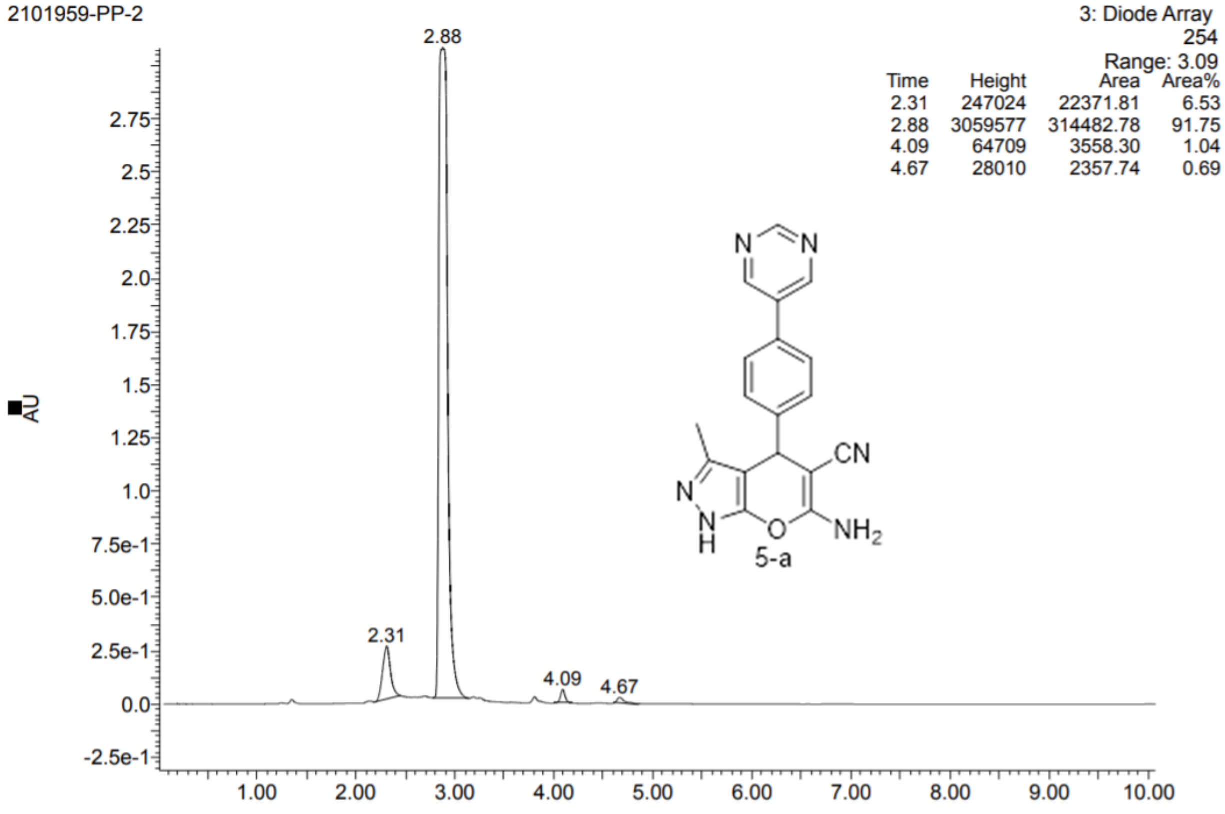

Lc of 5a

***

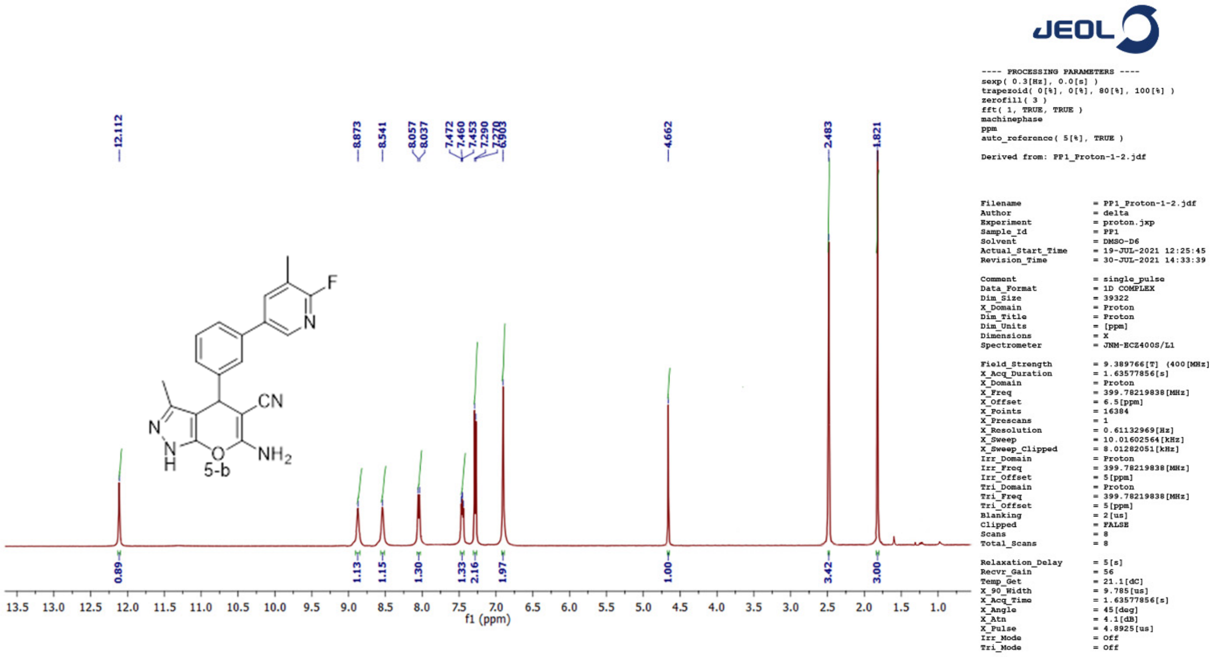

^{1}H NMR of 5b

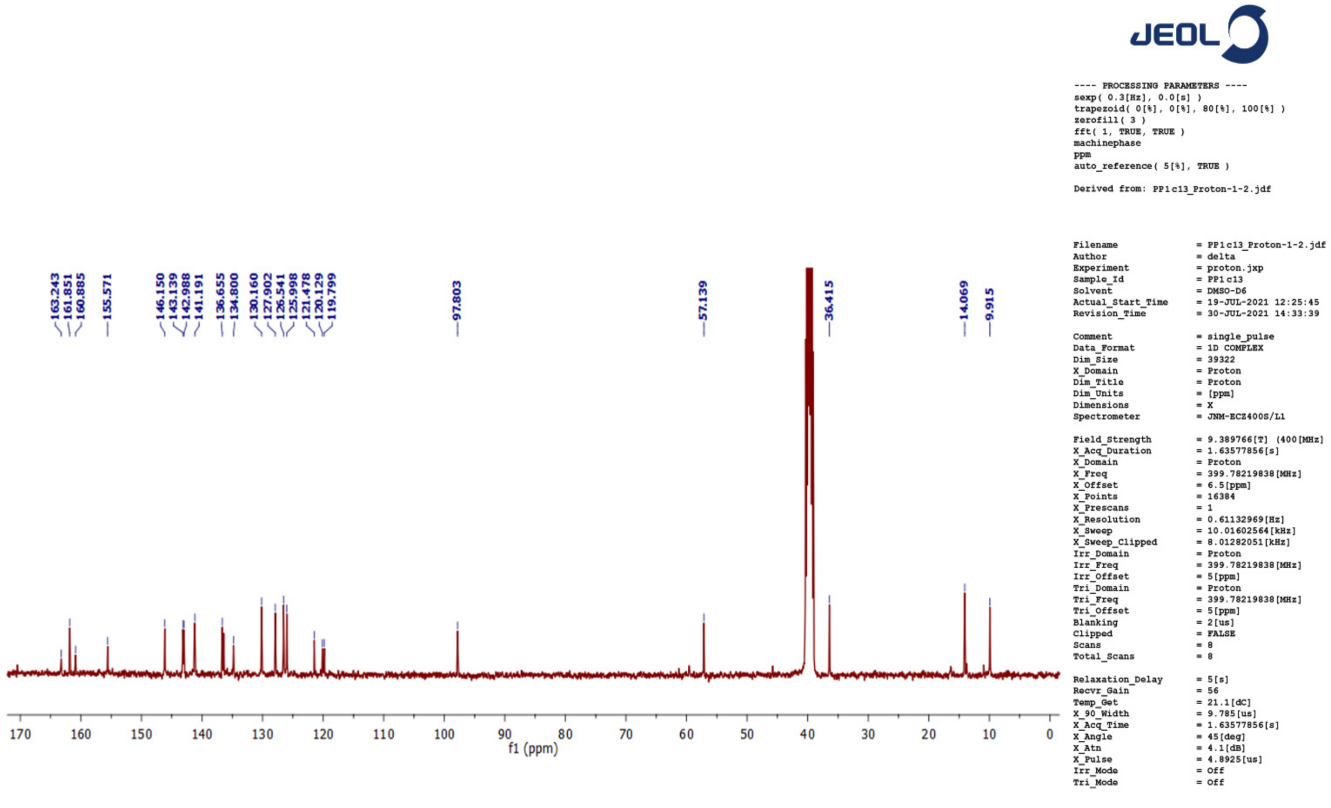

^{13}C NMR of 5-b

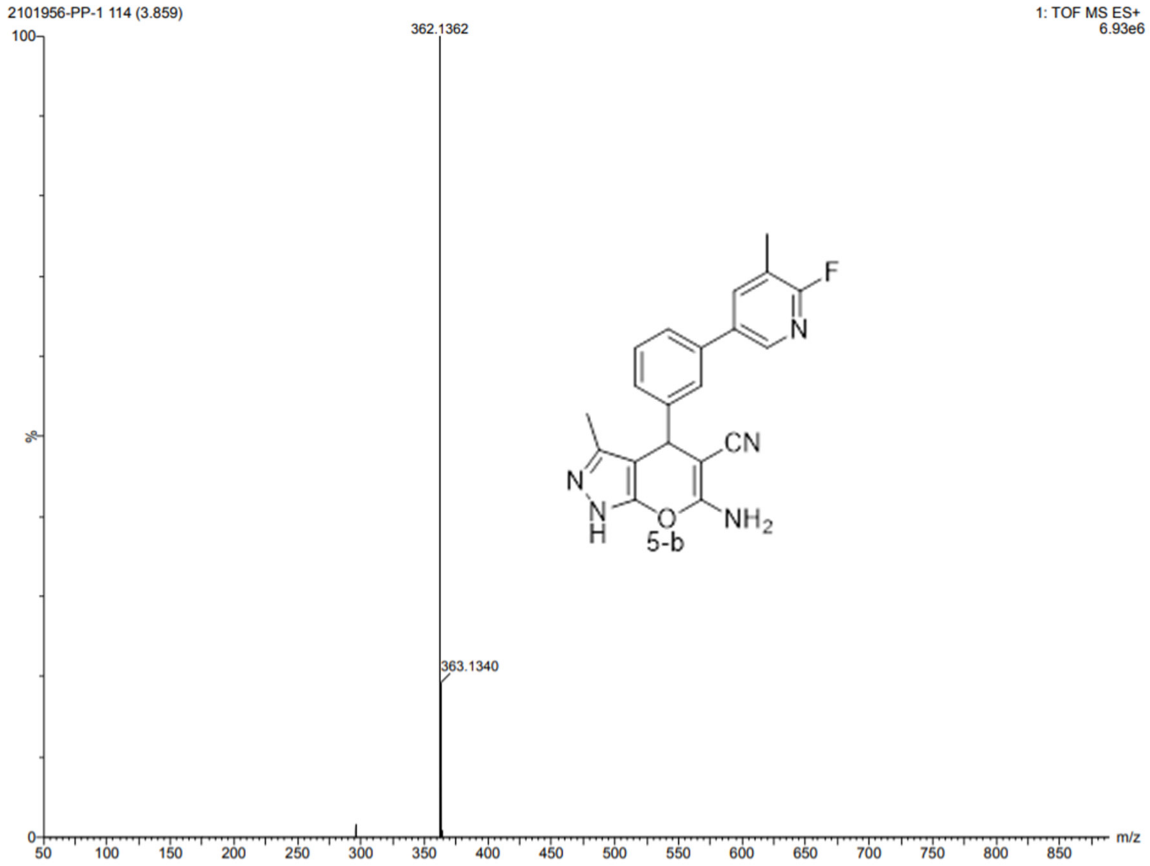

mass spectra of 5-b

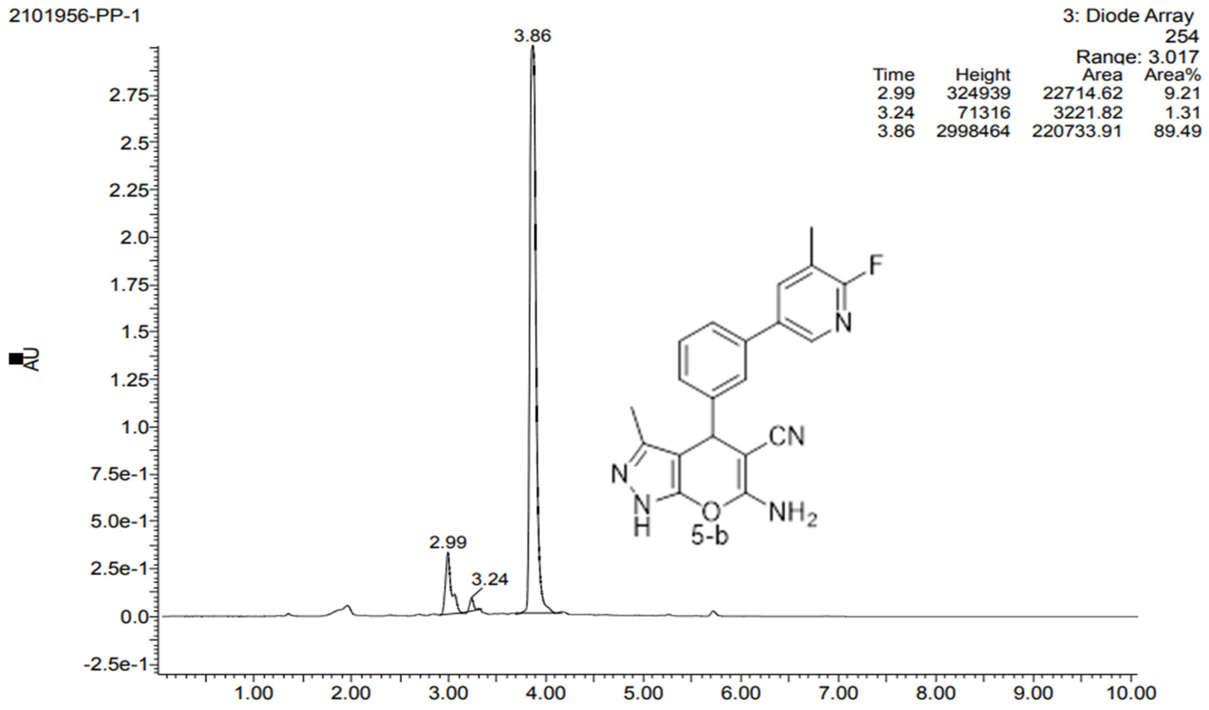

LC of 5-b

MCF-7 Log curve of 5-b

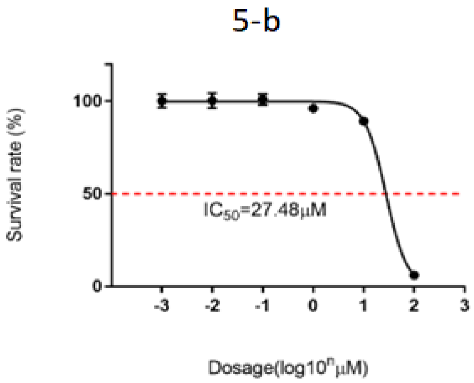

T47D log curve of 5-b

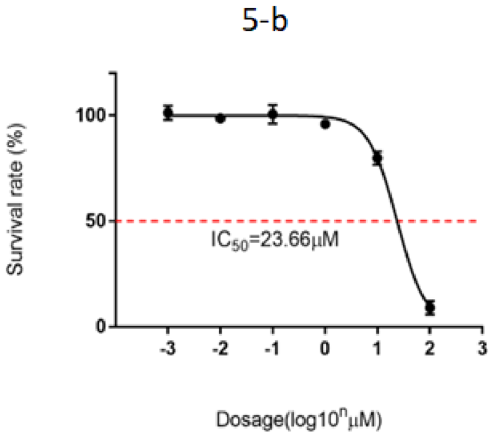

BT474 log curve of 5-b

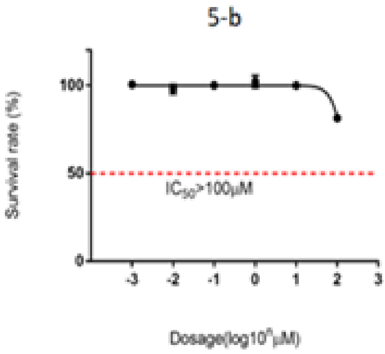

SKBR-3 log curve of 5-b

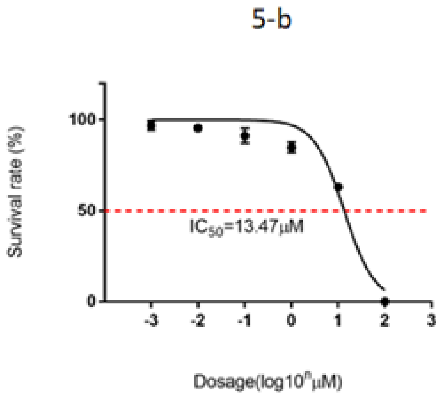

MDAMB-231 Log curve of 5-b

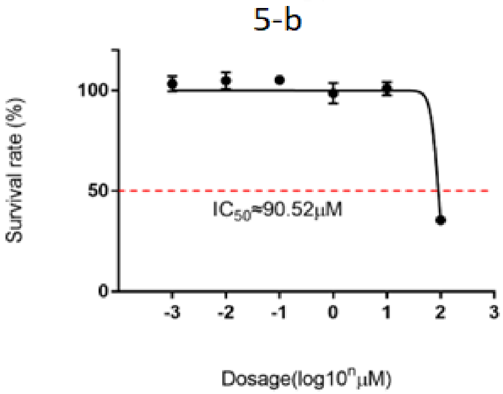

MCF-10A log curve of 5-b

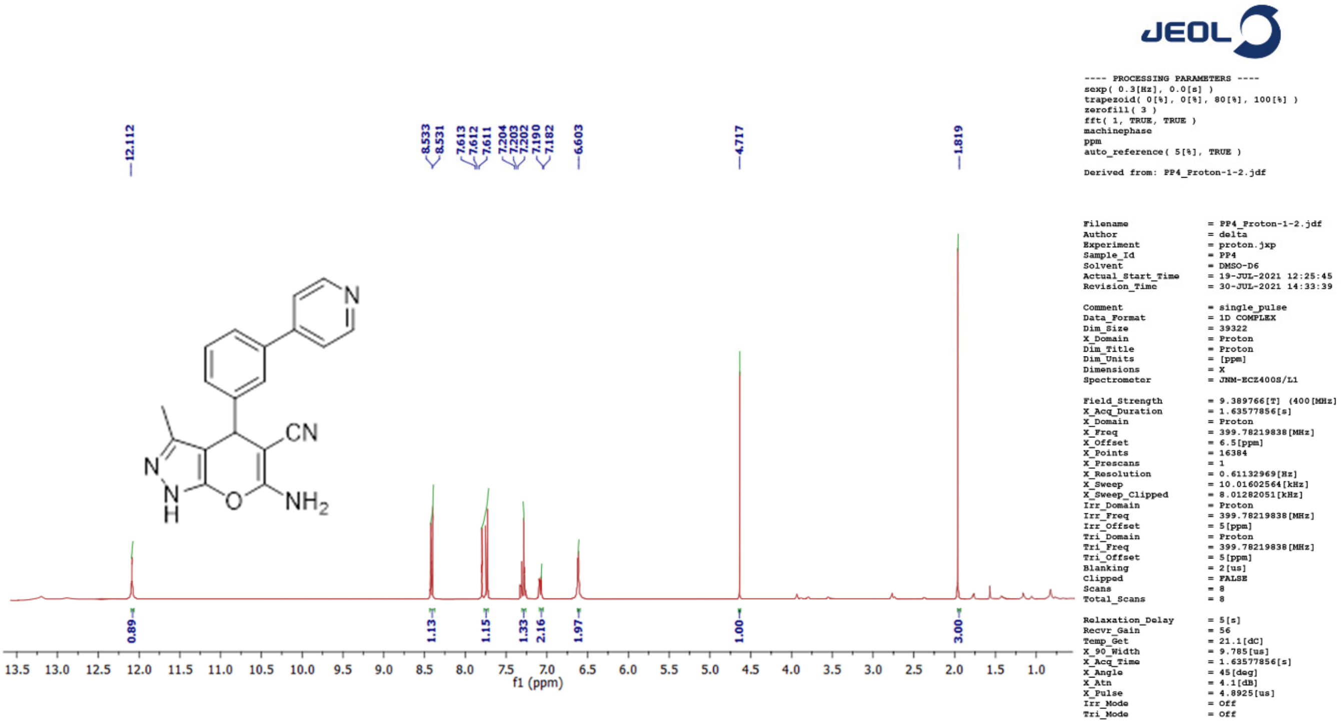

^{1}H NMR of 5-c

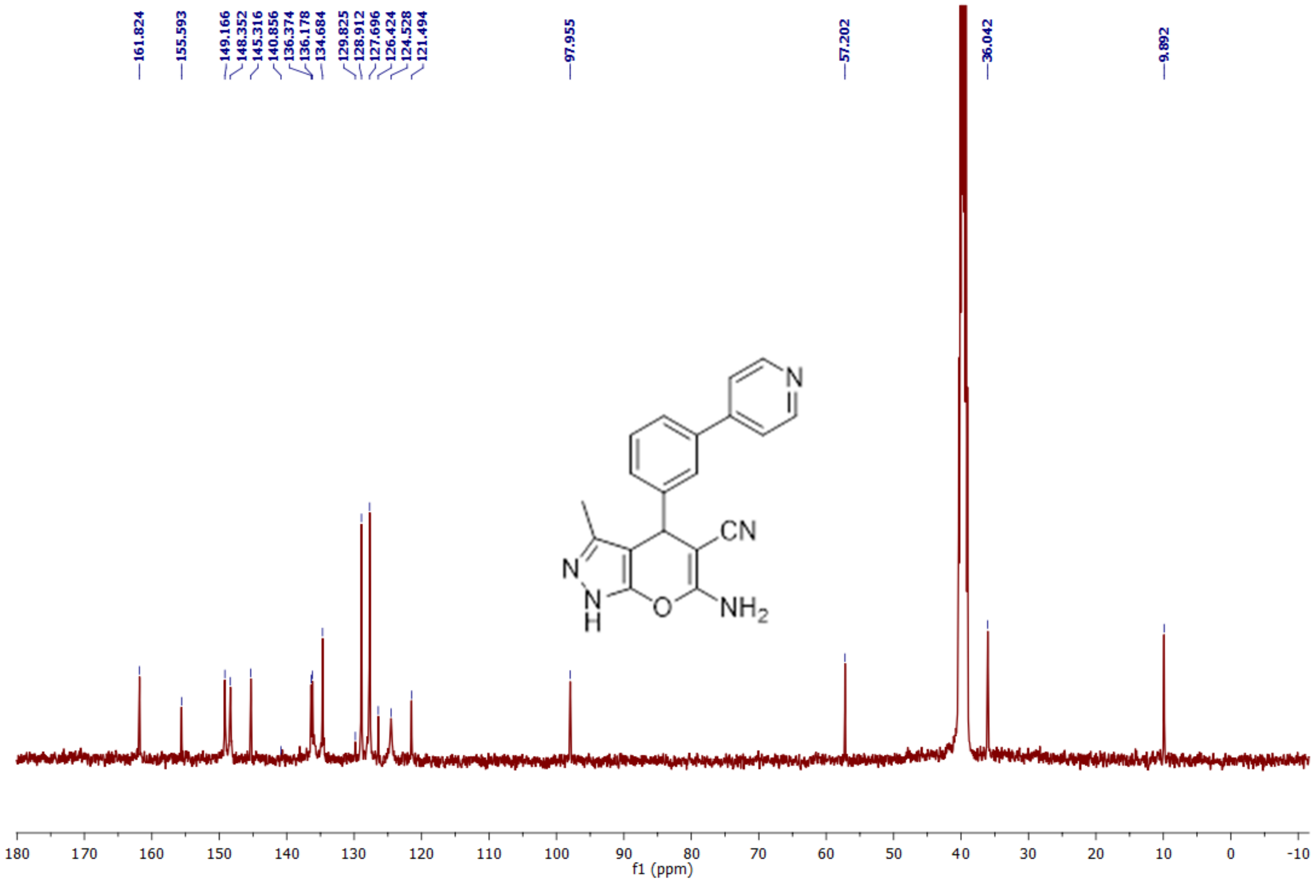

^{13}C NMR of 5-c

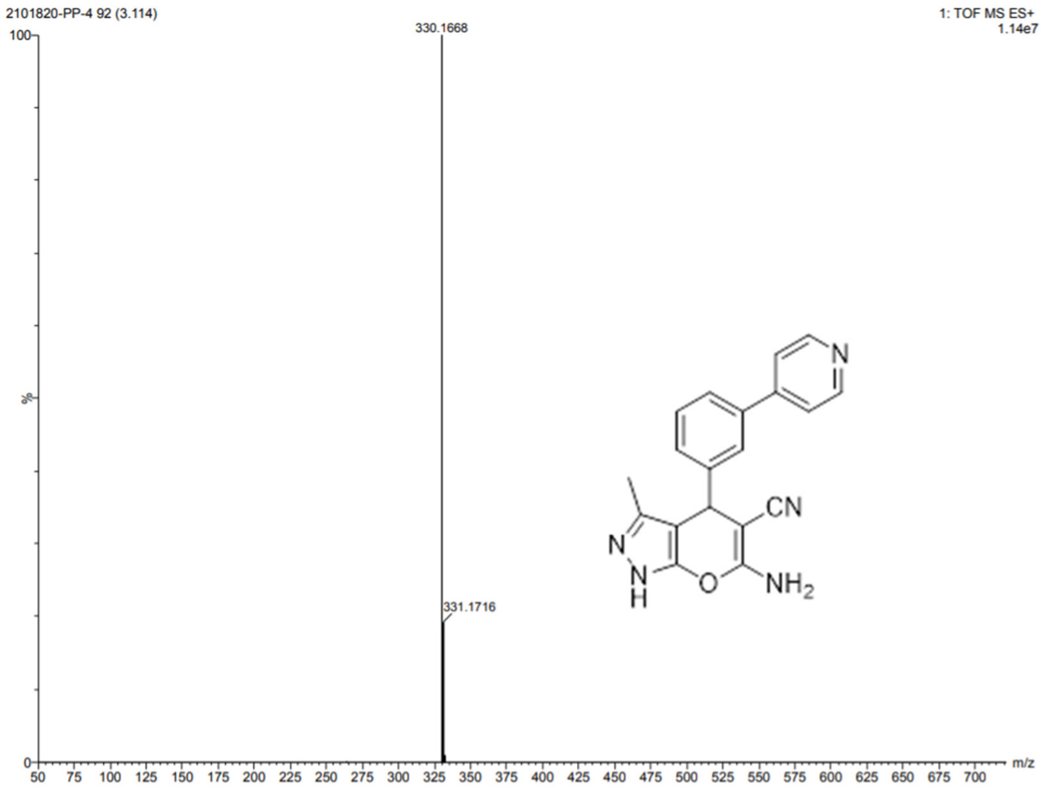

mass spectra of 5-c

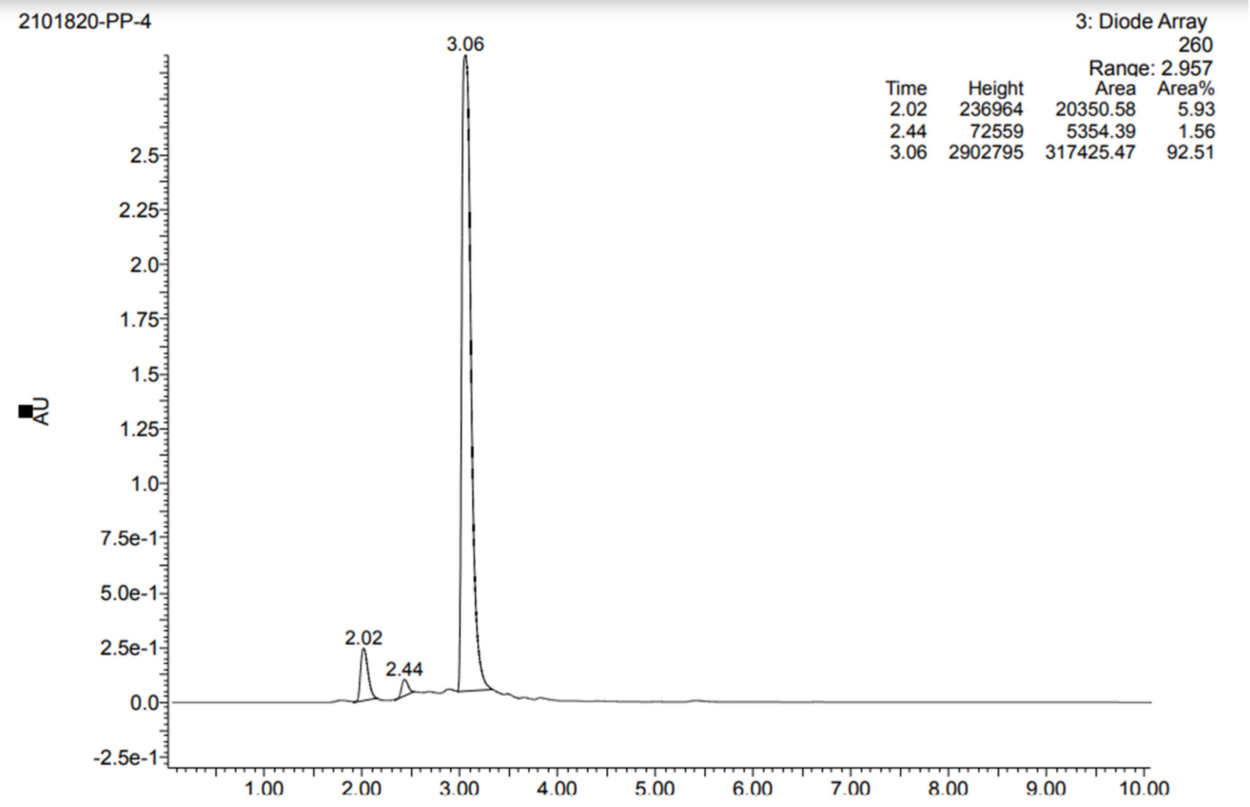

LC Of 5-c

MCF-7 log curve of 5-c

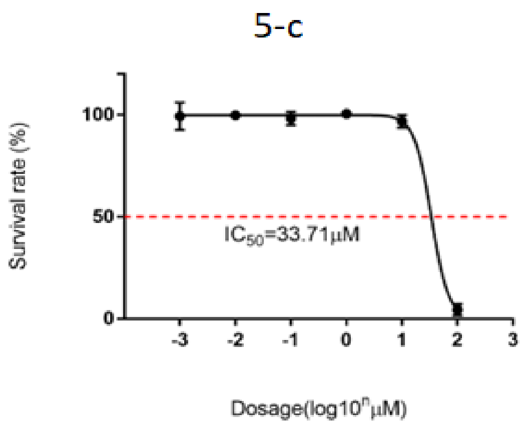

T47D log curve of 5c

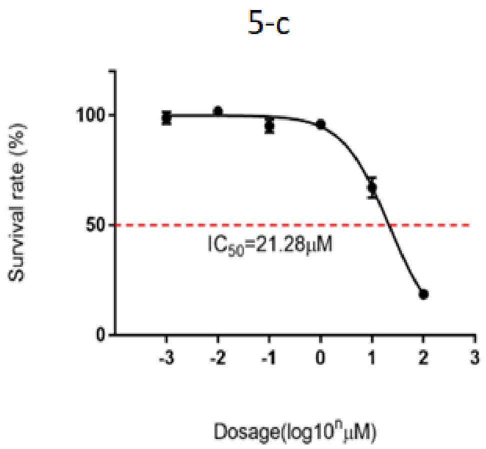

BT474 log curve of 5c

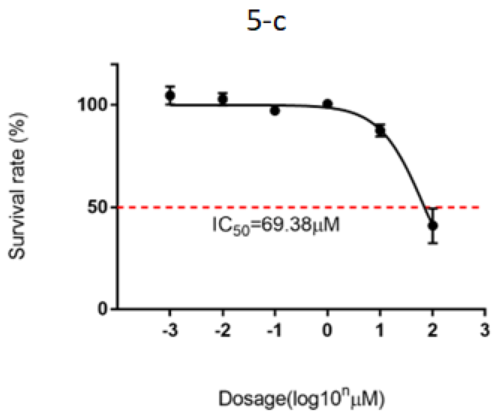

SKBR-3 log curve of 5-c

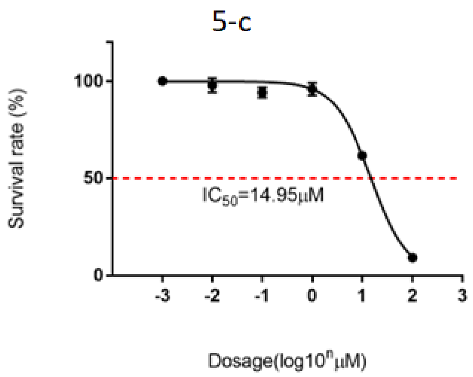

MDAMB-231 log curve of 5-c

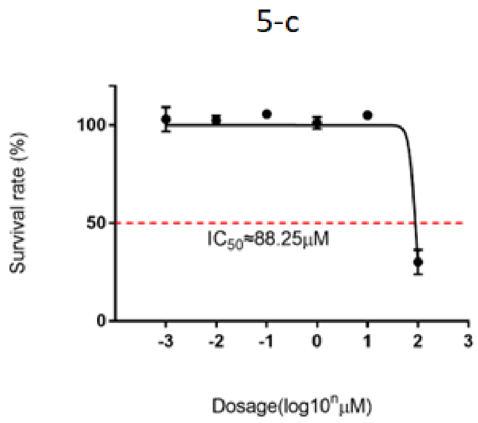

MCF-10A log curve of 5-c

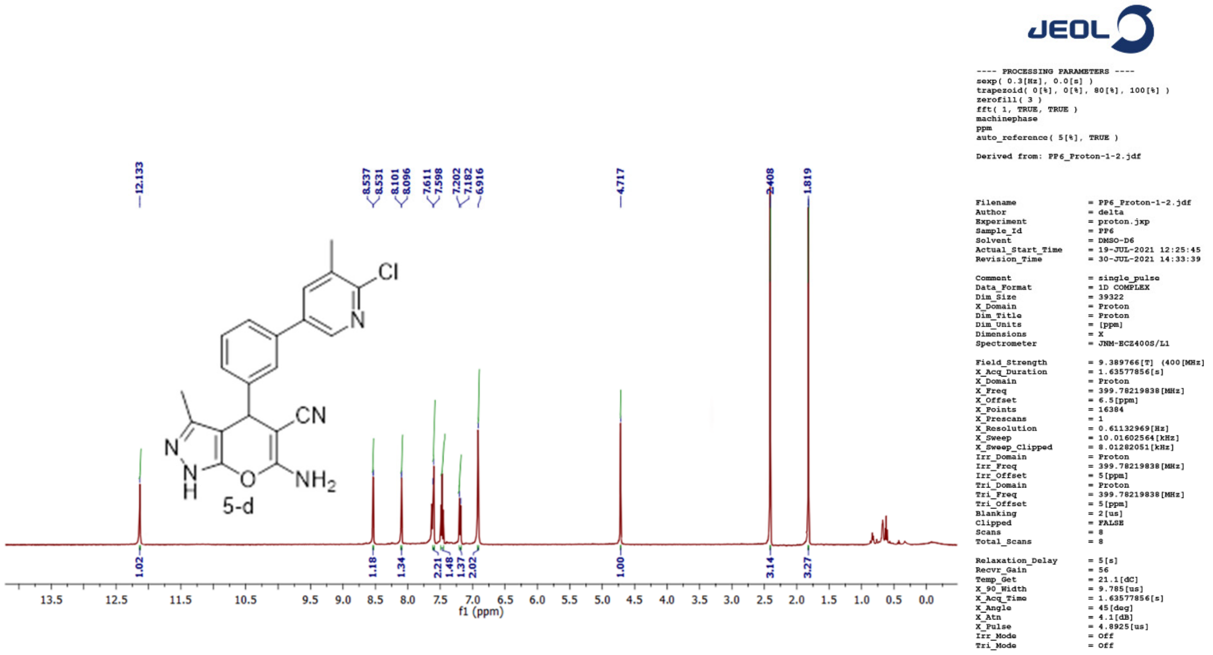

^{1}H NMR of 5-d

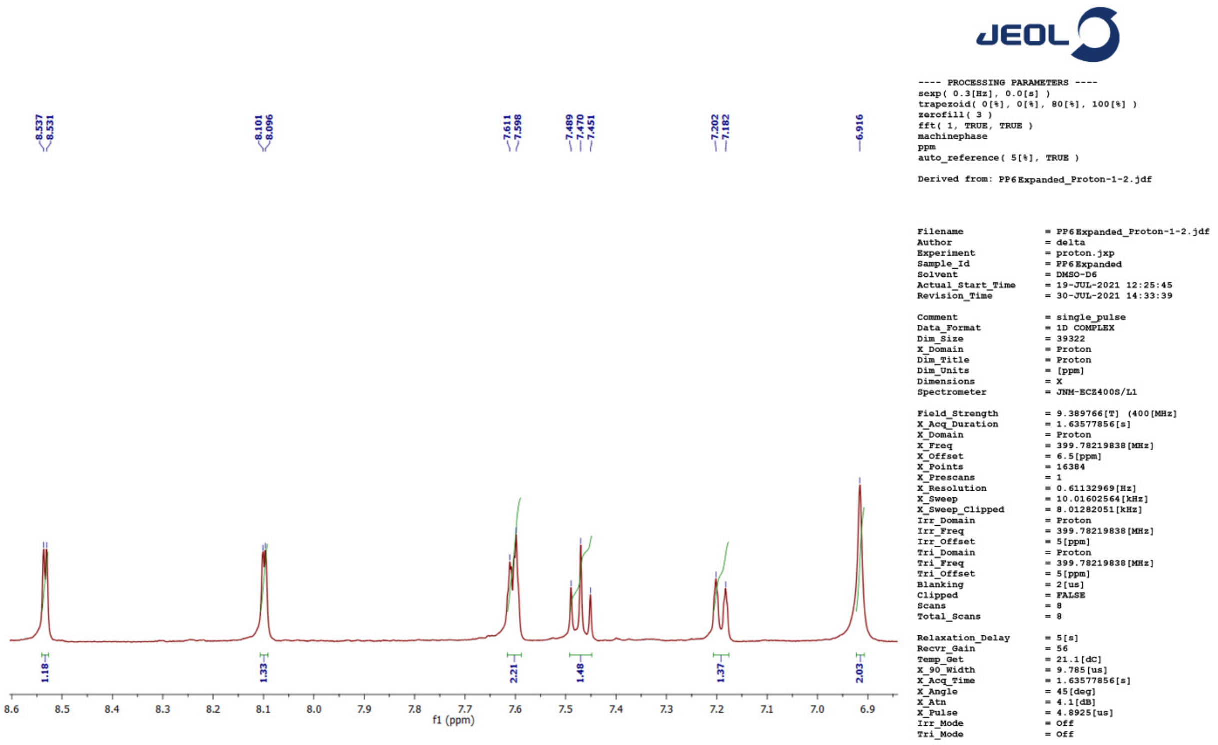

Expanded H NMR of 5d

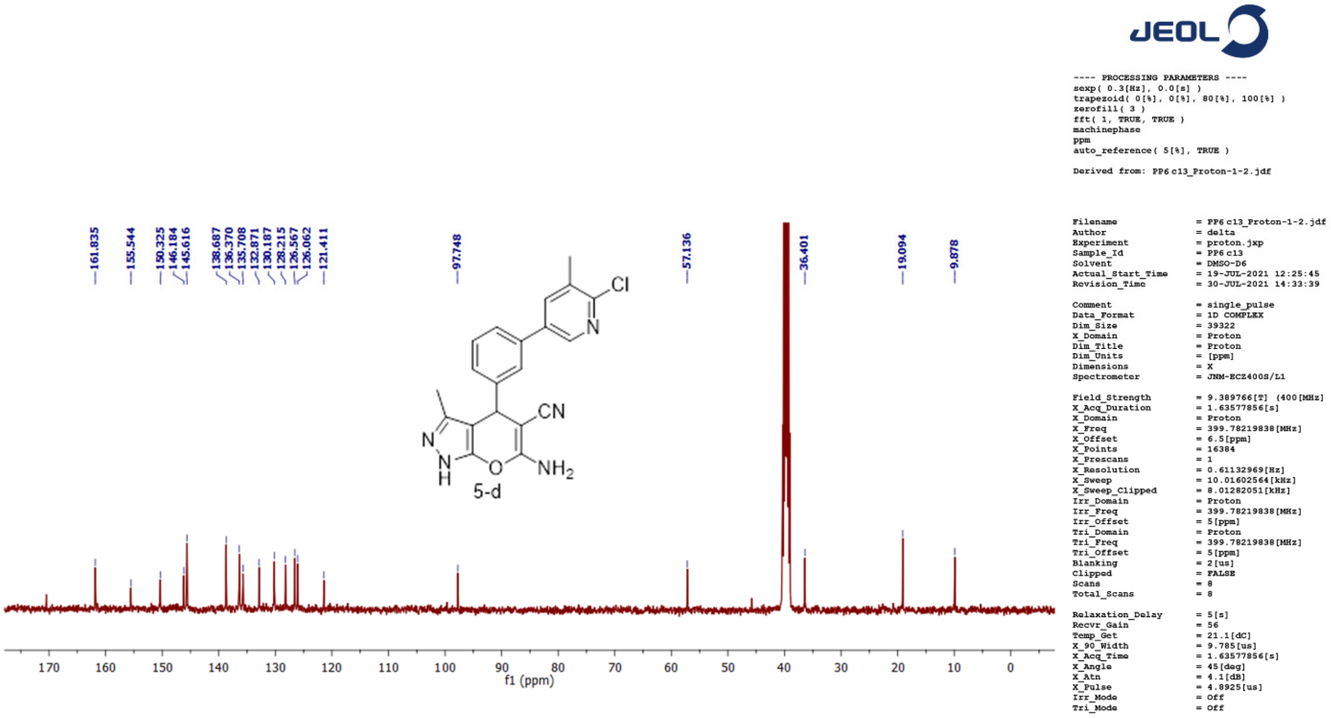

161.835
155.544
150.325
146.184
145.616
138.687
136.370
135.708
132.871
130.187
128.215
126.567
126.062
121.411
97.748
57.136
36.401
19.094
9.878
Cl
N
CN
N
N
H
O
NH2
5-d
170
160
150
140
130
120
110
100
90
80
70
60
50
40
30
20
10
0
f1 (ppm)
---- PROCESSING PARAMETERS ----
sexp(0.3[Hz], 0.0[s])
trapezoid(0[%], 0[%], 80[%], 100[%])
zerofill(3)
fft(1, TRUE, TRUE)
machinephase
ppm
auto_reference(5[%], TRUE)
Derived from: PP6 c13_Proton-1-2.jdf
Filename = PP6 c13_Proton-1-2.jdf
Author = delta
Experiment = proton.jxp
Sample_Id = PP6 c13
Solvent = DMSO-D6
Actual_Start_Time = 19-JUL-2021 12:25:45
Revision_Time = 30-JUL-2021 14:33:39
Comment = single_pulse
Data_Format = 1D COMPLEX
Dim_Size = 39322
X_Domain = Proton
Dim_Title = Proton
Dim_Units = [ppm]
Dimensions = X
Spectrometer = JNM-ECZ400S/L1
Field_Strength = 9.389766[T] (400[MHz]
X_Acq_Duration = 1.63577856[s]
X_Domain = Proton
X_Freq = 399.78219838[MHz]
X_Offset = 6.5[ppm]
X_Points = 16384
X_Prescans = 1
X_Resolution = 0.61132969[Hz]
X_Sweep = 10.01602564[kHz]
X_Sweep_Clipped = 8.01282051[kHz]
Irr_Domain = Proton
Irr_Freq = 399.78219838[MHz]
Irr_Offset = 5[ppm]
Tri_Domain = Proton
Tri_Freq = 399.78219838[MHz]
Tri_Offset = 5[ppm]
Blanking = 2[us]
Clipped = FALSE
Scans = 8
Total_Scans = 8
Relaxation_Delay = 5[s]
Recvr_Gain = 56
Temp_Get = 21.1[dC]
X_90_Width = 9.785[us]
X_Acq_Time = 1.63577856[s]
X_Angle = 45[deg]
X_Atn = 4.1[dB]
X_Pulse = 4.8925[us]
Irr_Mode = Off
Tri_Mode = Off

^{13}C NMR of 5-d

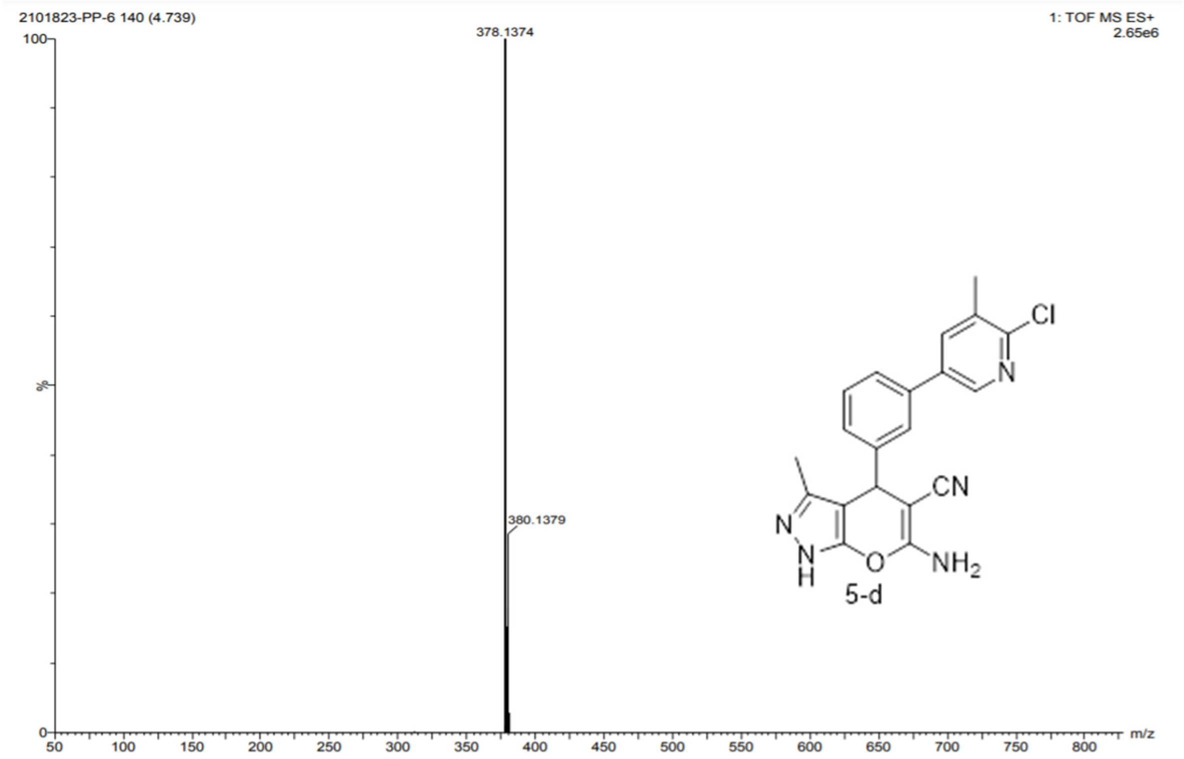

mass spectra of 5-d

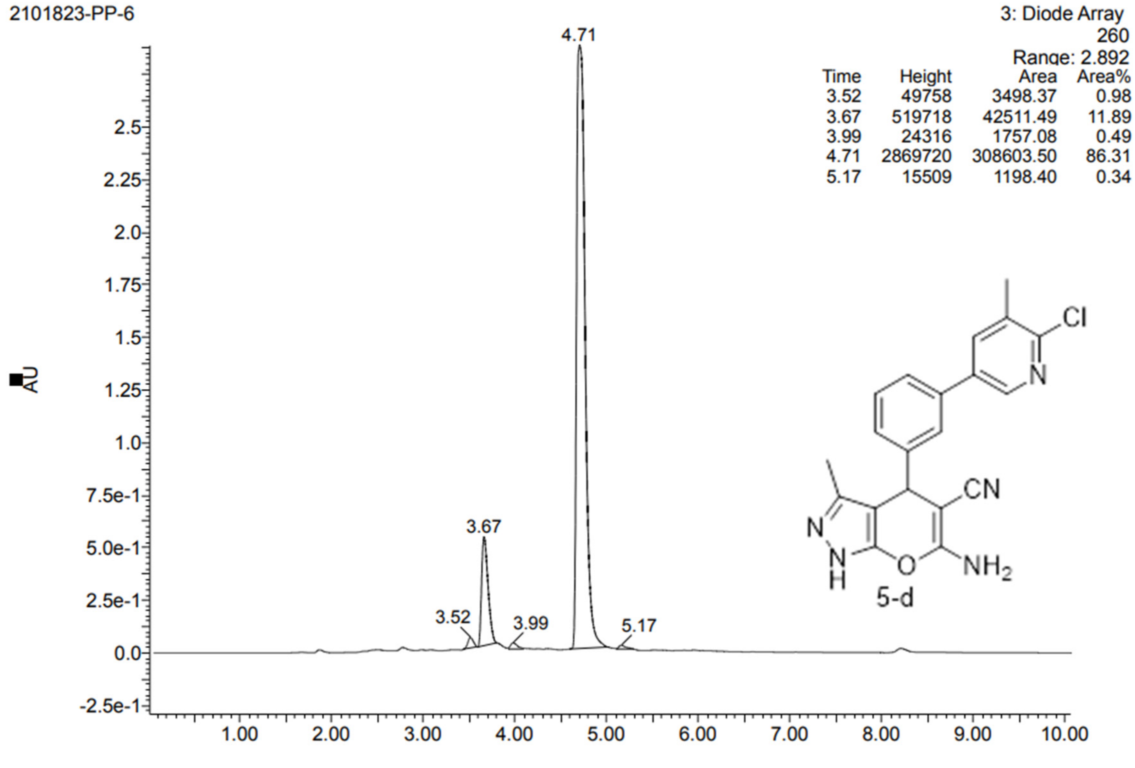

LC of 5d

***

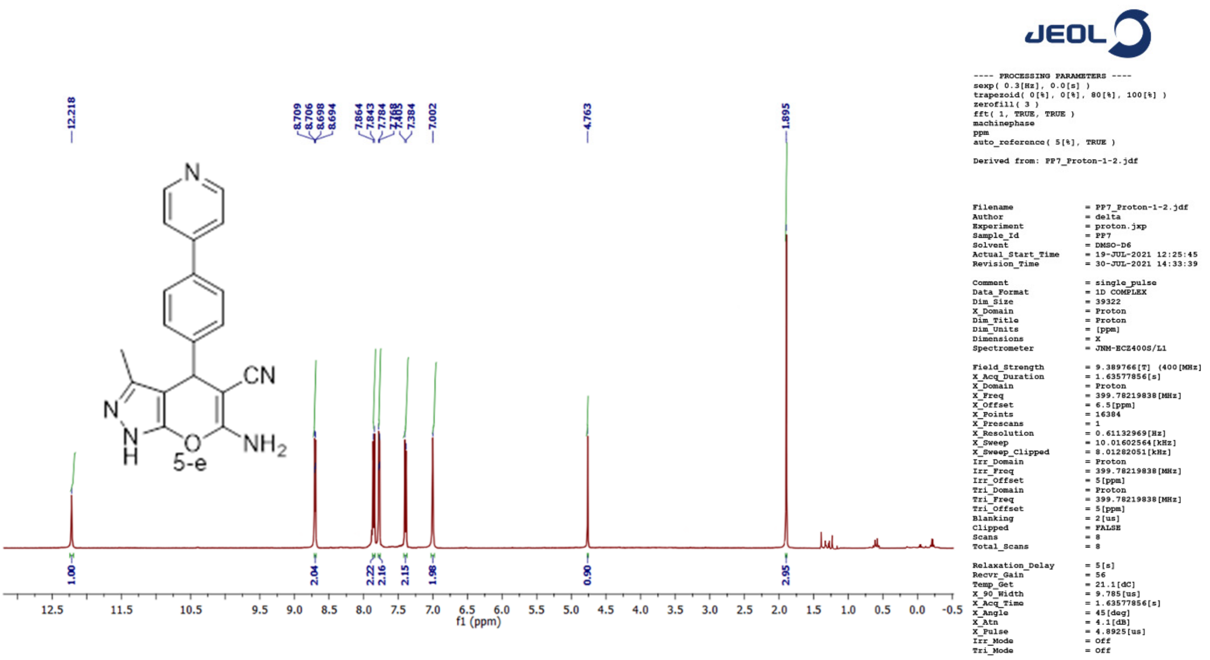

^{1}H NMR of 5-e

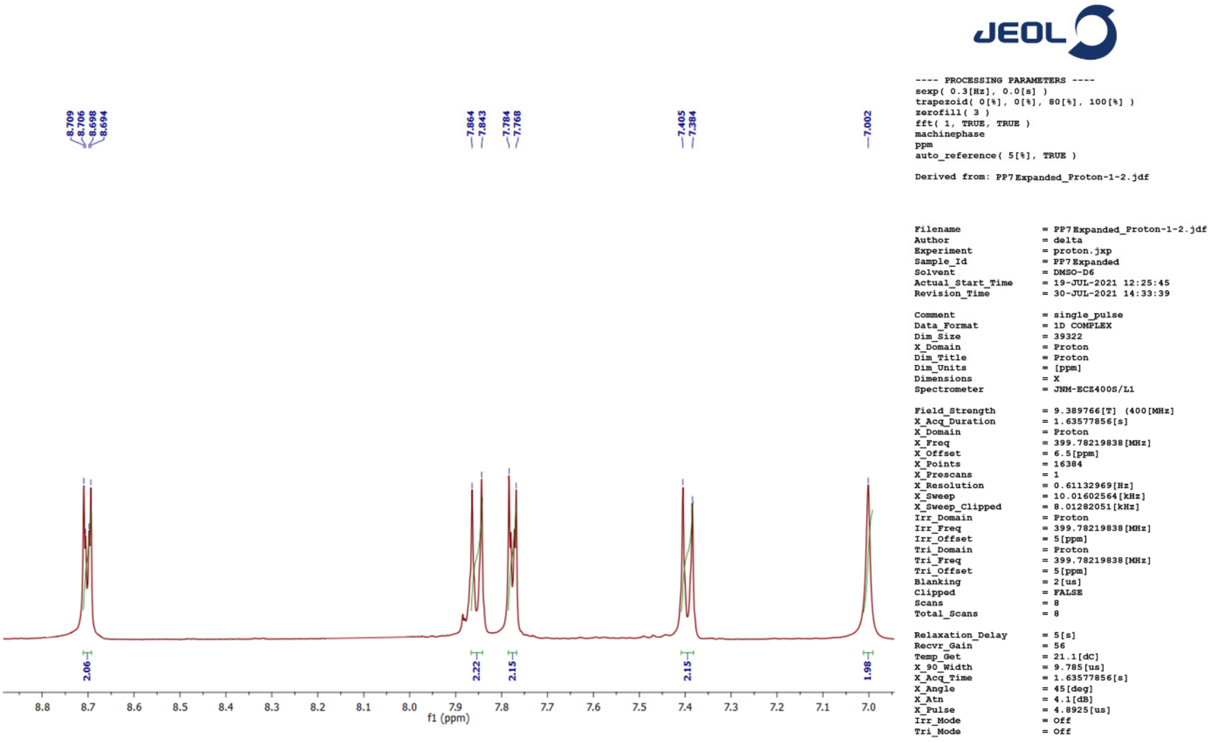

Expanded H NMR of 5e

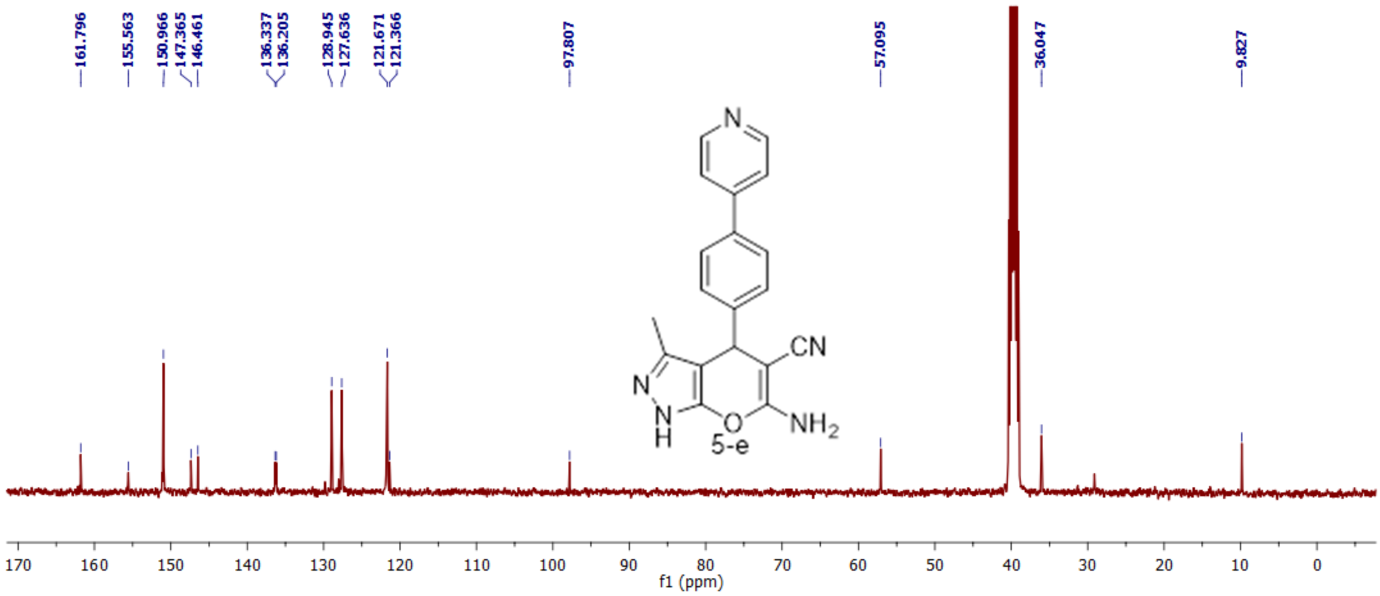
161.796
155.563
150.966
147.365
146.461
136.337
136.205
128.945
127.636
121.671
121.366
97.807
57.095
36.047
9.827
N
CN
N
N
H
O
NH2
5-e
f1 (ppm)

^{13}C NMR of 5-e

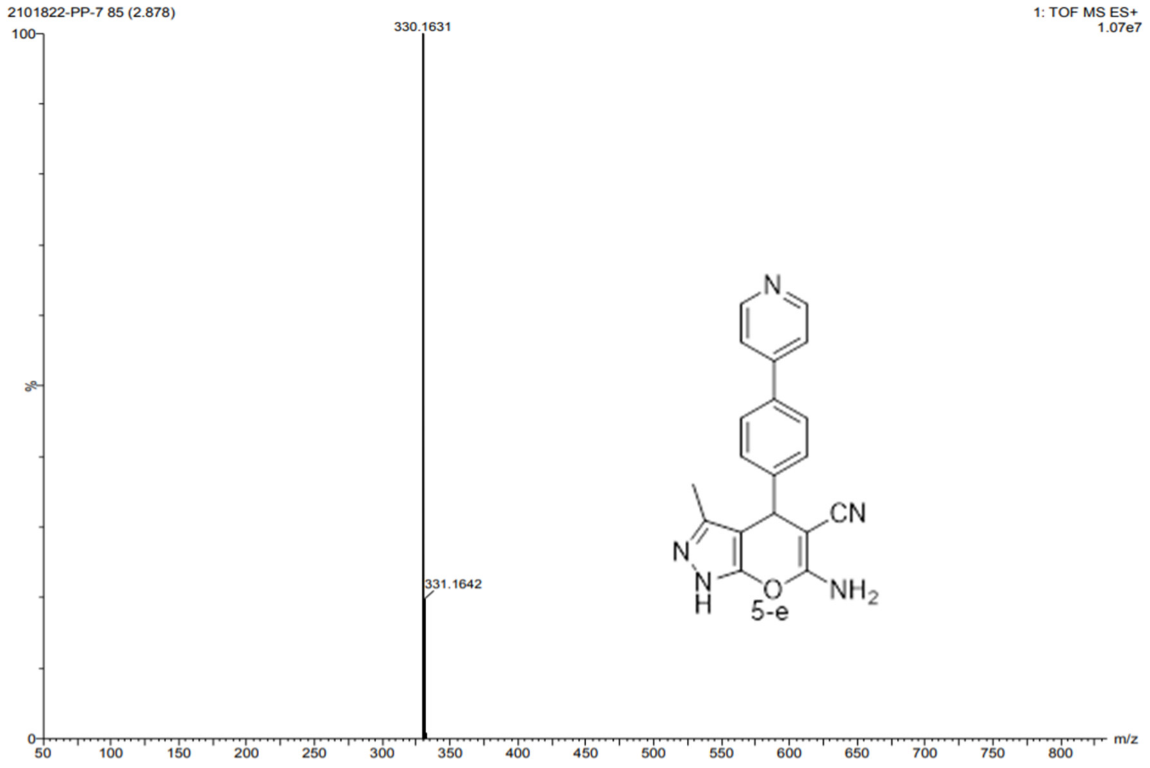

mass spectra of 5-e

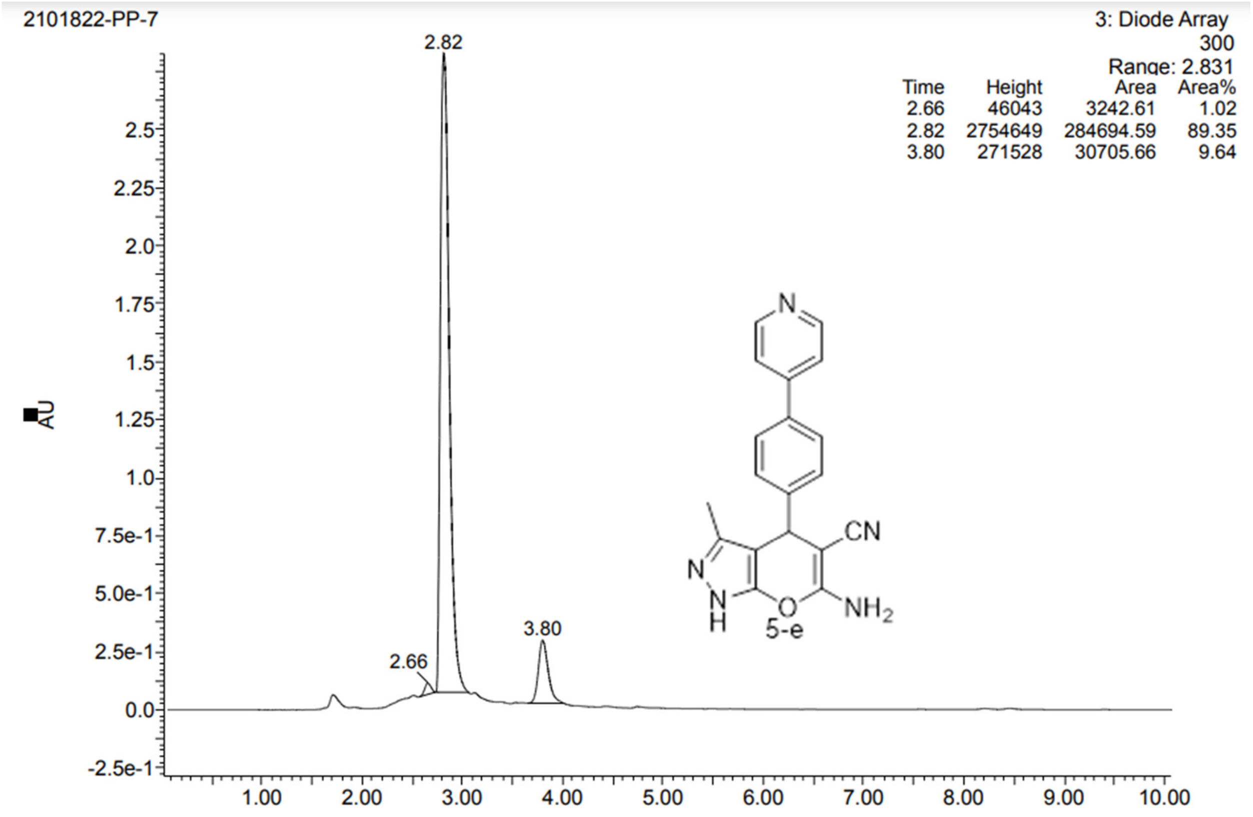

LC of 5-e

MCF-7 log curve of 5-e

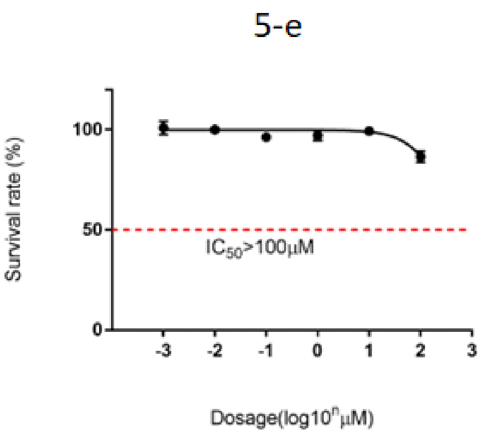

T47D log curve of 5-e

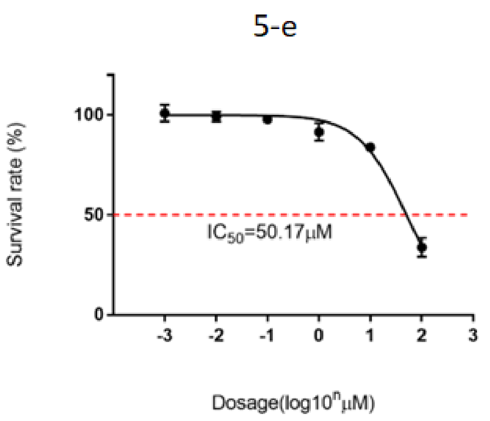

BT474 log curve of 5-e

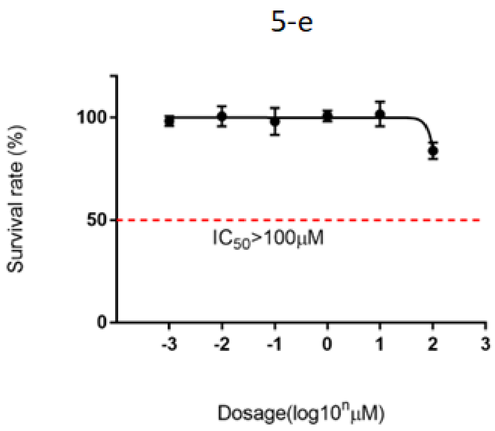

SKBR-3 log curve of 5-e

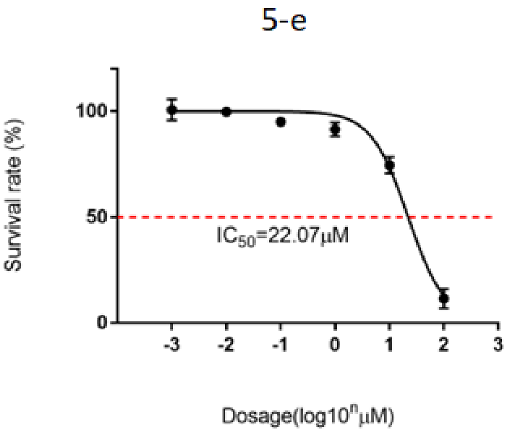

MDAMB-231 log curve of 5-e

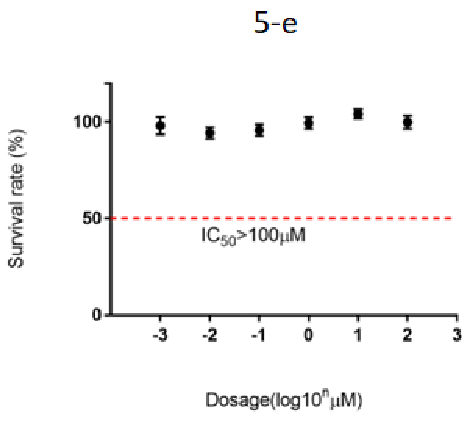

MCF-10A log curve of 5-e

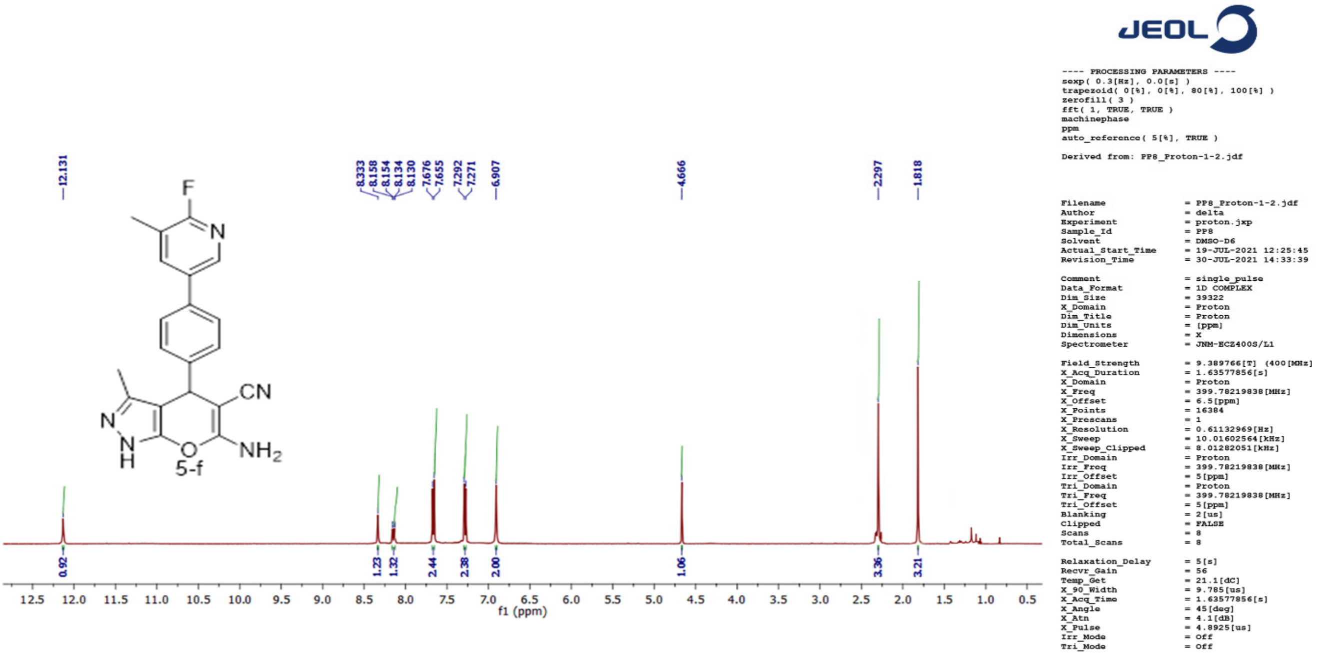

^{1}H NMR of 5-f

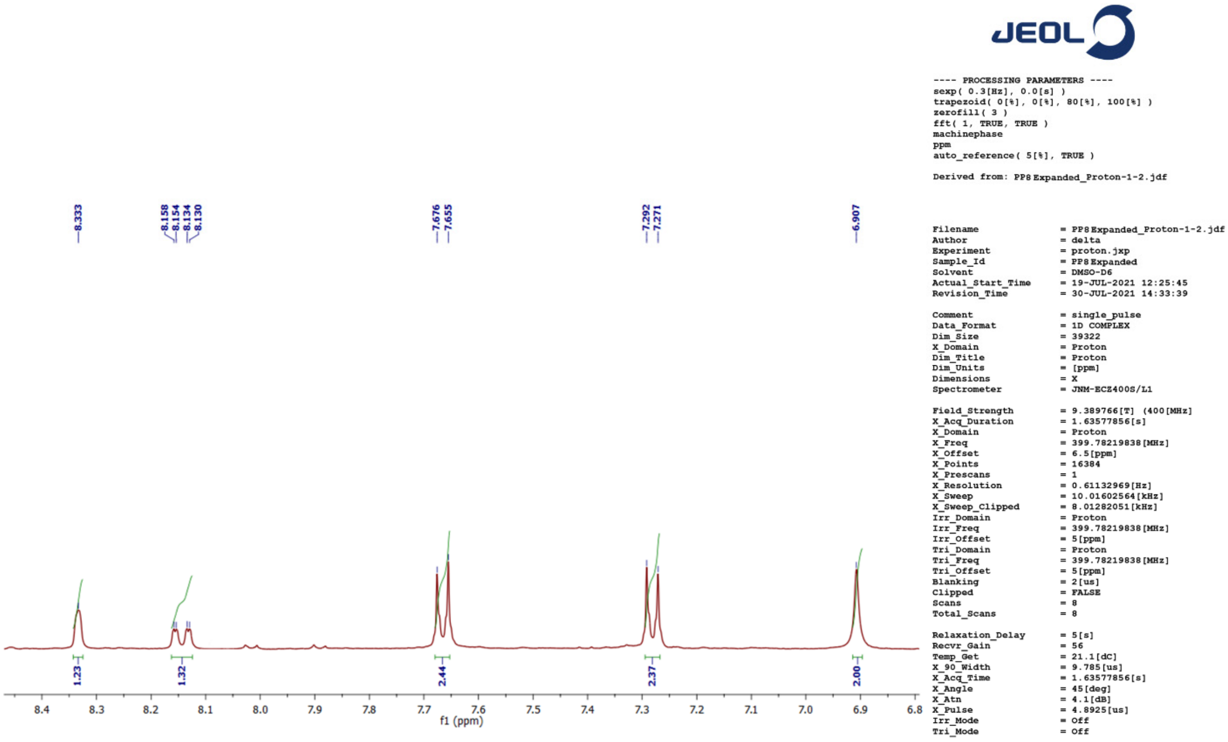

Expanded spectra of 5-f

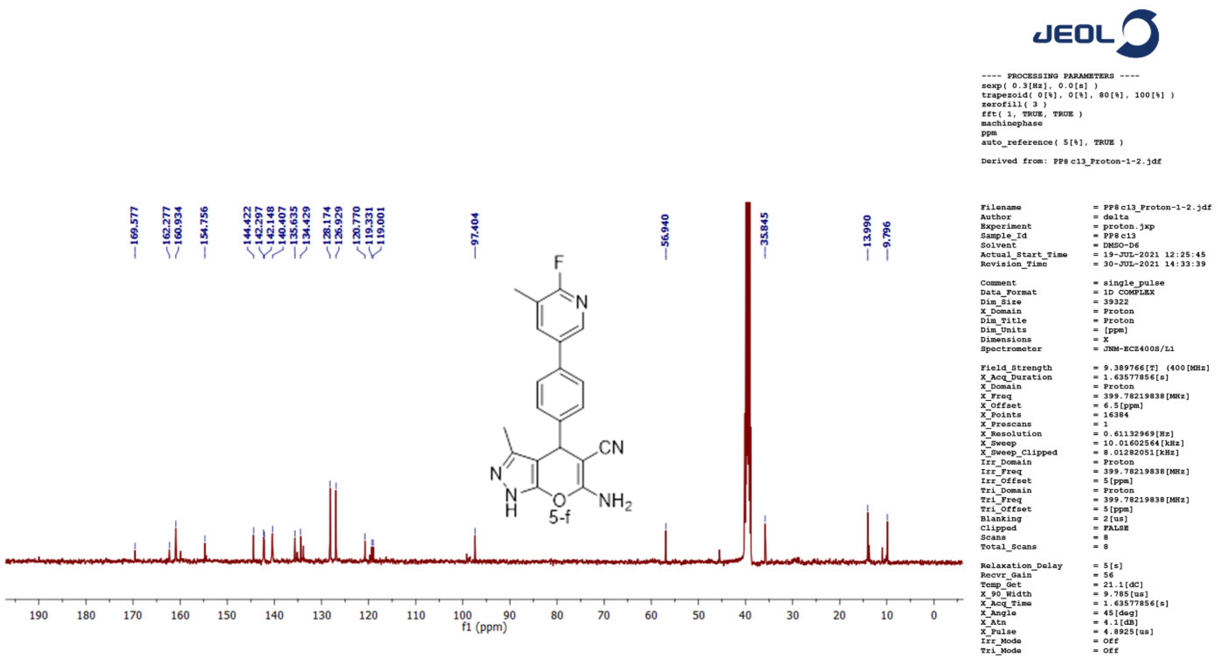

^{13}C NMR of 5-f

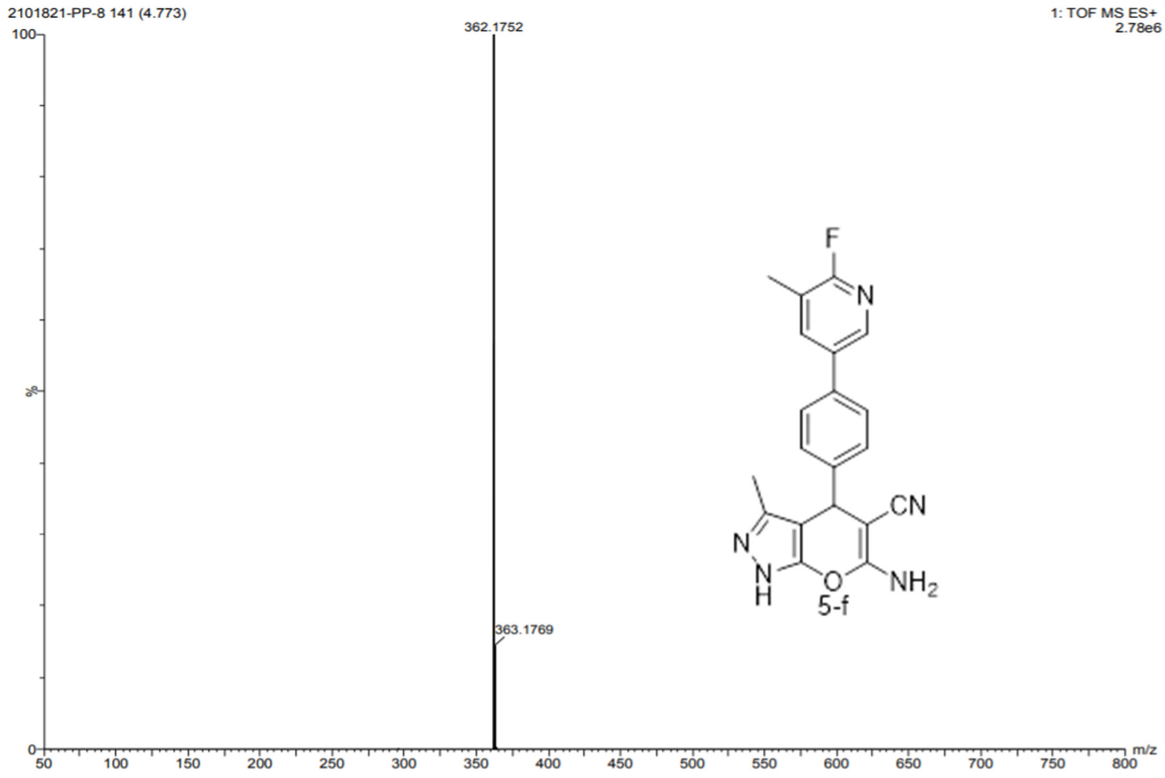

mass spectra of 5-f

MCF-7 log curve of 5-f

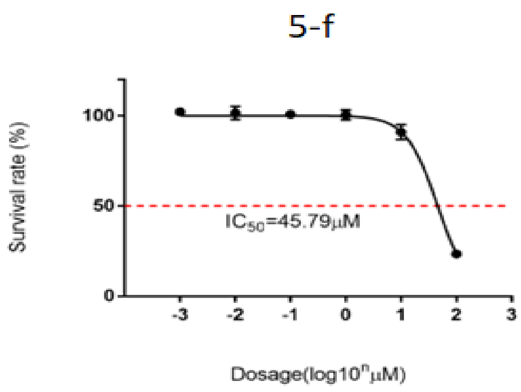

T47D log curve of 5-e

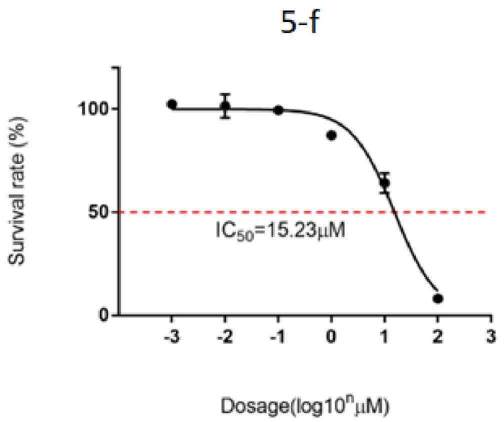

BT474 log curve of 5-e

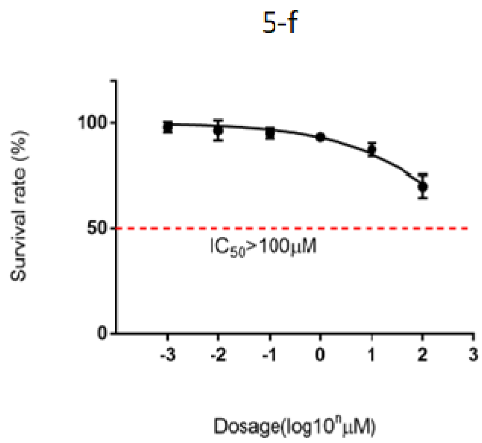

SKBR-3 log curve of 5-e

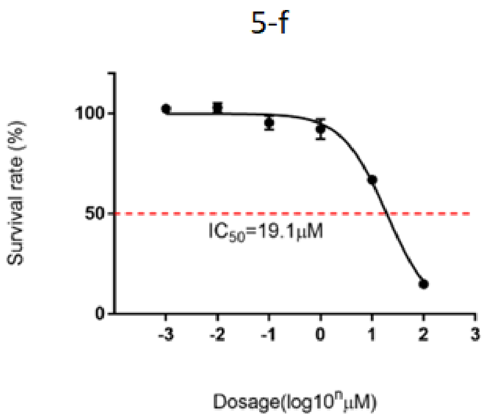

MDAMB-231 log curve of 5-e

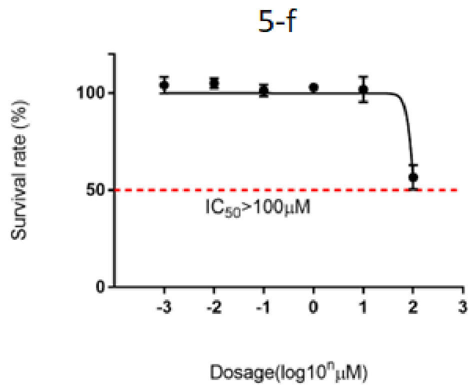

MCF-10A log curve of 5-e

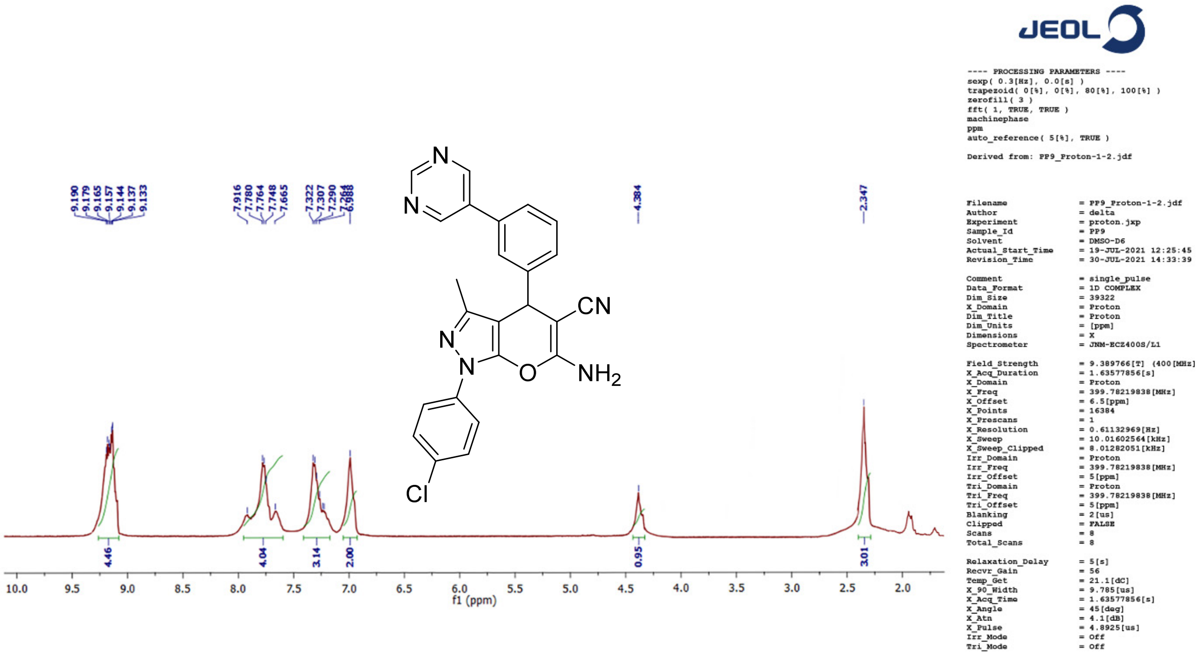

^{1}H NMR of 5g

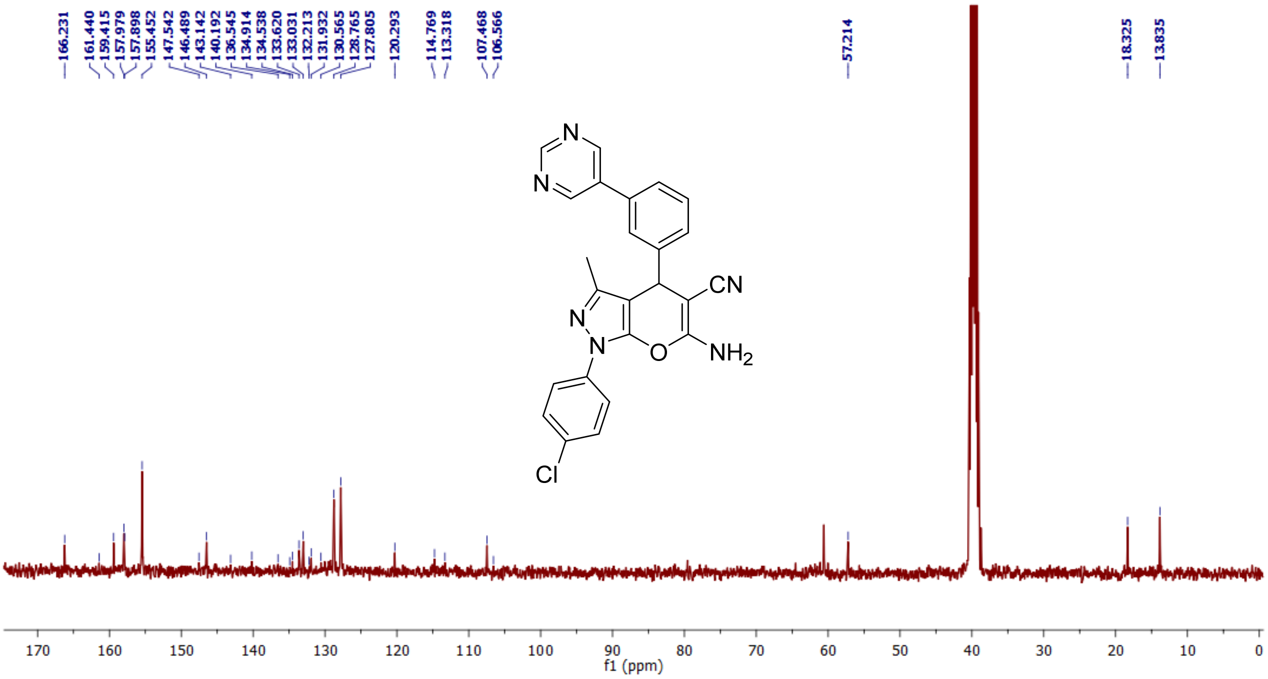

^{13}C NMR of 5g

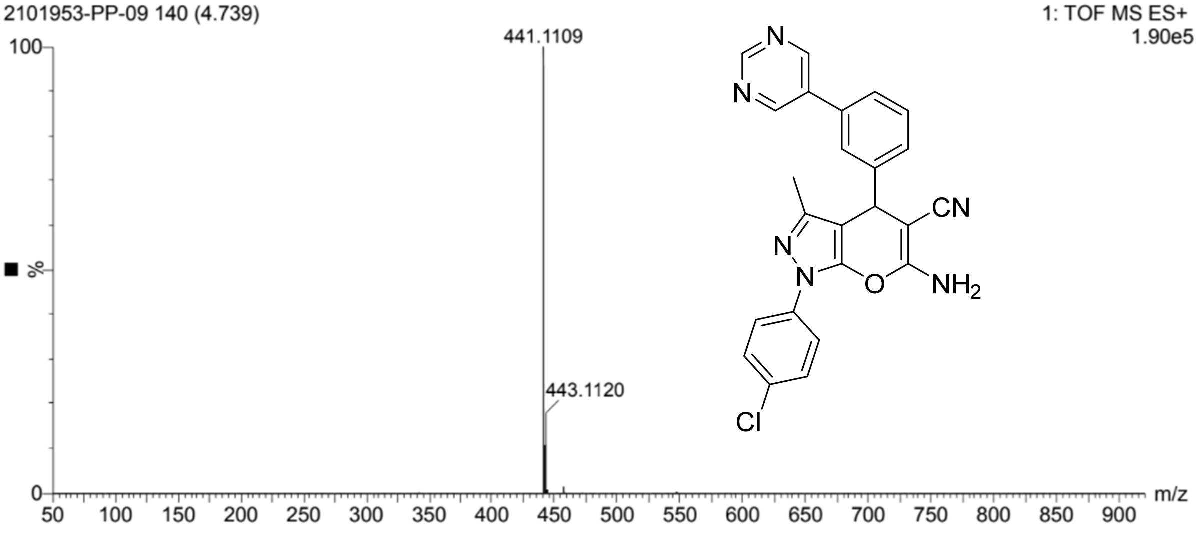

mass spectra of 5g
